# Bone Marrow‐Derived GCA^+^ Immune Cells Drive Alzheimer's Disease Progression

**DOI:** 10.1002/advs.202303402

**Published:** 2023-11-10

**Authors:** Rui Zhou, Liwen Wang, Linyun Chen, Xu Feng, Ruoyu Zhou, Peng Xiang, Jie Wen, Yan Huang, Haiyan Zhou

**Affiliations:** ^1^ Department of Endocrinology Endocrinology Research Center Xiangya Hospital of Central South University Changsha Hunan 410008 China; ^2^ National Clinical Research Center for Geriatric Disorders Xiangya Hospital Changsha Hunan 410008 China

**Keywords:** alzheimer's disease, bone marrow, GCA^+^ immune cells, LRP1

## Abstract

Alzheimer's disease (AD) is an age‐related degenerative disease of the central nervous system (CNS), whereas the role of bone marrow immune cells in the pathogenesis of AD remains unclear. Here, the study reveals that compared to matched healthy individuals, AD patients have higher circulating grancalcin (GCA) levels, which negatively correlate with cognitive function. Bone marrow‐derived GCA^+^ immune cells, which secret abundant GCA and increase during aging, preferentially invaded the hippocampus and cortex of AD mouse model in a C‐C Motif Chemokine Receptor 10 (CCR10)‐dependent manner. Transplanting GCA^+^ immune cells or direct stereotaxic injection of recombinant GCA protein intensified amyloid plaque load and aggravated cognitive and memory impairments. In contrast, genetic ablation of GCA in the hematopoietic compartment improves cognitive and memory function. Mechanistically, GCA competitively binds to the low‐density lipoprotein receptor‐related protein 1 (LRP1) in microglia, thus inhibiting phagocytosis and clearance of Aβ and potentiating neuropathological changes. Importantly, GCA‐neutralizing antibody treatment rejuvenated cognitive and memory function and constrained AD progression. Together, the study demonstrates a pathological role of GCA^+^ immune cells instigating cognitive and memory decline, suggesting that GCA^+^ immune cells can be a potential target for innovative therapeutic strategies in AD.

## Graphical abstract

The study shows bone marrow‐derived GCA^+^ immune cells secret abundant GCA and invade the brain via the CCR10‐CCL28 axis, thus aggravating the pathology of Alzheimer's disease (AD) in mice. Mechanistically, GCA competitively binds to LRP1 to inhibit Aβ clearance in microglia. Importantly, they demonstrate that GCA‐neutralizing antibody treatment alleviates cognitive decline and AD progression.

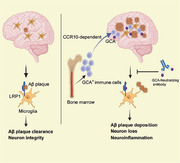

## 1 Introduction

Alzheimer's disease (AD) is a progressive, unremitting, neurodegenerative disease characterized by memory impairment and cognitive dysfunction.^[^ ^1^ ^]^ The prominent features of AD pathogenesis are extracellular amyloid β (Aβ) plaques and microtubule tau‐formed neurofibrillary tangles in neurons.^[^ ^1^ ^]^ Excessive Aβ accumulation is caused by the imbalance between neuronal Aβ production and extracellular Aβ clearance.^[^ ^2^ ^]^ While, Aβ aggregation triggers an immune response and contributes to microglia activation, which, as major resident immune cells in the brain, play a vital role in the clearance of Aβ and maintenance of the plasticity of neuron circuit, and axonal protection.^[^ ^3^ ^]^ Microglia losing the ability to clear misfolded proteins leads to neurodegeneration.^[^ ^4^ ^]^

The bone marrow is the primary reservoir of hematopoiesis, harboring hematopoietic stem cells, myeloid and lymphoid progenitors, as well as mature immune cells, including B cells, neutrophils, macrophages, and T cells.^[^ ^5^ ^]^ Previous studies have demonstrated that peripheral immune cells could naturally migrate into the brain and protect against Aβ accumulation and cognitive impairment.^[^ ^6^ ^]^ However, some studies found that the replacement of brain‐resident myeloid cells with peripheral myeloid cells is insufficient to alter Aβ deposition.^[^ ^7^ ^]^ Additionally, strategies to restore the metabolism of myeloid cells reverse cognitive decline in aging mice.^[^ ^8^ ^]^ These researches raise a key research point that various peripheral immune cells may play a specific role in Aβ burden clearance and AD progression.

Grancalcin (GCA) belongs to the penta‐EF‐hand Ca^2^^+^‐binding protein family,^[^ ^9^ ^]^ which is implicated in the regulation of cell migration, apoptosis, and mobilization of immune cells.^[^ ^10^ ^]^ We have previously reported that senescent GCA^+^ immune cells secrete plentiful GCA to repress osteogenesis, while *Gca* deletion in myeloid cells ameliorates skeletal aging.^[^ ^11^ ^]^ As AD is also an aging‐related disease, it is interesting to speculate that GCA^+^ immune cells may also play a vital role in AD progression.

In this study, we leveraged a single‐cell database and found that Gca was highly expressed in the circulating macrophages in AD patients. We verified that GCA^+^ immune cells greatly accumulated in the brain of AD mice. Adoptive transfer of bone‐marrow derived GCA^+^ immune cells into AD animal model clarified that these cells preferentially migrated into the brain via the C‐C Motif Chemokine Receptor 10 (CCR10)‐C‐C Motif Chemokine Receptor 10 (CCL28) axis and accelerated AD development. Intracerebral injection of GCA recombinant protein aggravated cognitive impairment and the pathogenesis of AD. By contrast, *Gca‐VAV1‐CKO* mice displayed obviously improved cognitive dysfunction. Mechanistically, GCA could competitively bind with LRP1 in microglia cells to restrain Aβ clearance. Importantly, we developed a GCA‐neutralizing antibody and rendered that its treatment ameliorates cognitive impairment. Together, these observations uncover a novel mechanism whereby bone marrow GCA^+^ immune cells initiate cognitive decline, offering a novel therapeutic target for AD progression.

## 2 Results

### 2.1 Bone Marrow‐Derived GCA^+^ Immune Cells Accumulated in the Brain During AD Progression

**Figure 1:**
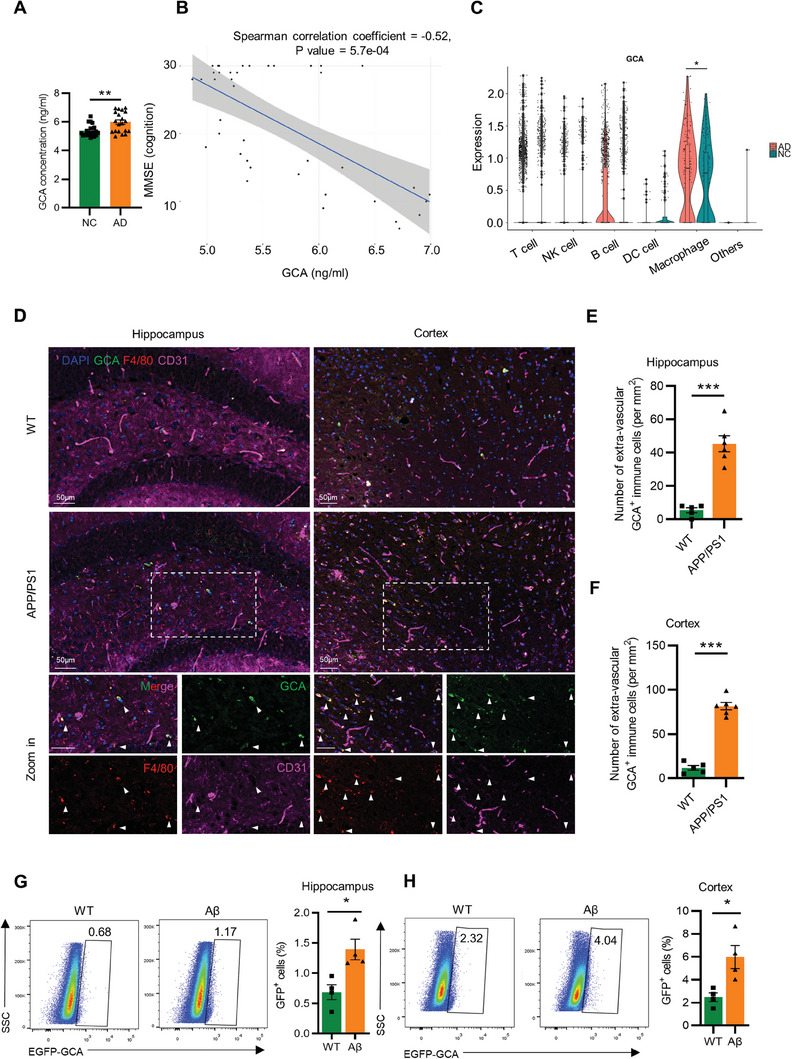
Bone marrow‐derived GCA^+^ immune cells accumulated in the brain during AD progression. A) ELISA analysis of the GCA concentration in peripheral blood from AD patients and NC (cognitively normal controls) (*n* = 20). B) Spearman correlation between the plasma concentration of GCA and MMSE score from AD patients and NC. C) Violin plots of log‐transformed gene expression of GCA in cell populations of peripheral blood immune cells collected from AD patients and NC. D) Representative higher magnification images of GCA (green), F4/80 (red), CD31 (violet), and nucleus (blue) staining in hippocampus and cortex. The boxed regions correspond to the zoomed‐in sections. White arrows indicate cells within the brain parenchyma. (Scale bar = 50 µm; *n* = 5–6). E) The number of extra‐vascular GCA^+^ immune cells (per mm^2^) in the hippocampus from WT and 6‐month‐old APP/PS1 mice (n = 5–6). F) The number of extra‐vascular GCA^+^ immune cells (per mm^2^) in the cortex from WT and 6‐month‐old APP/PS1 mice (*n* = 5–6). G) Representative flow cytometry plots and quantification of the frequencies of peripherally‐derived GCA^+^ immune cells in hippocampus from Aβ‐injected *Gca‐Cre‐EGFP* mice or control mice (*n* = 4). H) Representative flow cytometry plots and quantification of the frequencies of peripherally‐derived GCA^+^ immune cells in cortex from Aβ‐injected *Gca‐Cre‐EGFP* mice or control mice (*n* = 4). Data are shown as the mean ± SEM. For panel (A,E–H): unpaired, two‐tailed Student's t‐test. For panel (B): Spearman Correlation Analysis. For panel (C):Wilcoxon rank‐sum test. ^*^ *p* < 0.05, ^**^ *p* < 0.01, ^***^ *p* < 0.001.

To verify the relationship between GCA and AD, we first collected the peripheral blood samples of 20 AD patients and 20 age‐ and gender‐ matched healthy individuals (Table S1, Supporting Information). We found that the level of GCA protein in the peripheral blood of AD patients was significantly higher than that in healthy individuals, without gender differences (**Figure** **1**A; Table S2, Supporting Information). Moreover, there was a negative correlation between GCA concentration and the scores of Mini‐Mental State Examination (MMSE) tests, a standard clinical tool for measuring cognitive impairment (Figure 1B), indicating that GCA might play a role in the AD progression. To determine which circulating immune cells contributed to the increased GCA level, we further leveraged a single‐cell RNA sequencing dataset (GSE181279) that have analyzed circulating immune cells in healthy and AD patients.^[^ ^12^ ^]^ Notably, we found that GCA was most abundantly expressed in macrophages (Figure 1C; Figure S1A, Supporting Information), which was consistent with our previous results.^[^ ^11^ ^]^ The expression of GCA was even higher in macrophages from AD patients than in healthy individuals (Figure 1C).

The infiltration of peripheral immune cells in the brain has long been thought to be closely related to the pathogenesis of AD.^[^ ^13^ ^]^ Immunofluorescent staining showed increased infiltration of peripherally‐derived GCA^+^F4/80^hi^ immune cells in the hippocampus and cortex of APP/PS1 mice compared to normal control mice (Figure 1D–F). To further investigate the potential involvement of brain vascular immune cells in the increased GCA expression in the brain of APP/PS1 mice, we performed immunofluorescent staining of CD31, the endothelial cell marker, with GCA^+^ immune cells in the brain. The GCA^+^ immune cells mainly distributed in the brain parenchyma but less in the vascular compartment (Figure 1D; Figure S1B,C, Supporting Information). To further demonstrate a direct infiltration of bone‐marrow GCA^+^ immune cells, we generated a *Gca‐Cre‐EGFP* mouse model (Figure S1D,E, Supporting Information), which allows GCA^+^ immune cells to be identified as EGFP^+^ immune cells. We injected Aβ into the hippocampus of *Gca‐Cre‐EGFP* mice to establish an AD mouse model.^[^ ^14^ ^]^ We verified that Aβ oligomerization was formed during incubation period in vitro, and Aβ level in Aβ injection‐induced AD mice model was comparable to that in 6‐month‐old APP/PS1 mice. (Figure S1F,G, Supporting Information). To determine whether GCA^+^ immune cells truly infiltrated the brain parenchyma or blood contaminants, mice received intravenous injection of PE–conjugated anti‐CD45 antibody, which labeled blood and vascular‐associated leukocytes that were subsequently excluded in our gating strategy (Figure S1H, Supporting Information). Flow cytometry results indicated a significant increase in peripherally‐derived GCA^+^ immune cells (CD45^hi^CD11b^+^EGFP^+^) in the hippocampus and cortex of Aβ‐injected *Gca‐Cre‐EGFP* mice (Figure 1G,H). Together, these results suggest that peripherally‐derived GCA^+^ immune cells accumulate in the brain during AD progression.

### 2.2 Adoptive Transfer of GCA^+^ Immune Cells Aggravated Cognitive and Memory Impairments

**Figure 2:**
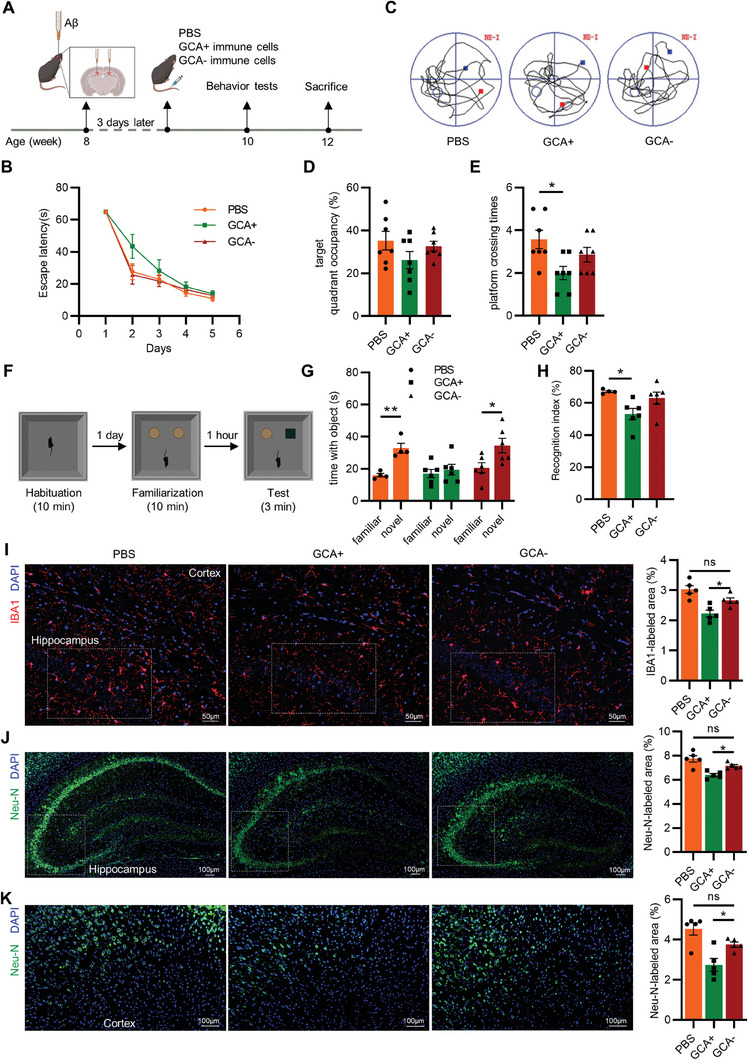
Adoptive transfer of GCA^+^ immune cells aggravated cognitive and memory impairments. A) Experiment design about GCA^+^ immune cells, GCA^−^ immune cells and PBS controls transferred into Aβ‐injected mice. B–E) In the MWM test, B) the escape latency time, C) representative motion trajectories, D) target quadrant occupancy, E) platform crossing times in GCA^+^ immune cells, GCA^−^ immune cells and PBS control groups (*n* = 7). F) Experiment design about novel object recognition test (NORT). G,H) Time with the object was assessed G) and recognition index H) of the NORT (*n* = 4–6). I) Representative images of IBA1 (red) and nucleus (blue) staining and quantification of IBA1 labeled area in hippocampus and cortex. The boxed regions correspond to Figure S2F. (Scale bar = 50 µm; *n* = 5). J) Representative images of Neu‐N (green) and nucleus (blue) staining in hippocampus and quantification of Neu‐N labeled area. The boxed regions correspond to Figure S2F (Scale bar = 100 µm; *n* = 5). K) Representative images of Neu‐N (green) and nucleus (blue) staining in cortex and quantification of Neu‐N labeled area. (Scale bar = 100 µm; *n* = 5). Data are shown as the mean ± SEM. For panel (B): two‐way ANOVA. For panel (D,E,H–K): one‐way ANOVA. For panel (G): paired, two‐tailed Student's t‐test. ^*^ *p* < 0.05, ^**^ *p* < 0.01, ^***^ *p* < 0.001.

To further investigate an initial role of GCA^+^ immune cells in the progression of AD, we conducted a cell transplantation experiment, as shown in **Figure** **2**A. GCA^+^ (CD11b^+^ Ly6C^+^Ly6G^−^EGFP^+^) and GCA^−^ (CD11b^+^Ly6C^+^ Ly6G^−^ EGFP^−^) immune cells were sorted from the bone marrow of *Gca‐Cre‐EGFP* mice by flow cytometry and labeled with lipophilic membrane dye PKH26 (Figure S2A, Supporting Information). After intracerebral injection of Aβ to induce AD progression, GCA^+^ immune cells, GCA^−^ immune cells, or PBS were intravenously injected into 2‐month‐old mice, followed by behavior tests (Figure 2A).

Two weeks after injection, the Morris water maze (MWM) and novel object recognition test (NORT) were used to evaluate cognitive function. Compared with the PBS or GCA^−^ immune cells group, the GCA^+^ immune cells‐treated mice were less efficient at finding the hidden platform and showed less platform crossing times (Figure 2B–E; Figure S2B, Supporting Information), indicating a deficit in spatial learning ability. No significant differences were found in mean swimming speed among three groups during the MWM (Figure S2C, Supporting Information). In the NORT, the time spent by the mice treated with familiar objects and novel objects was recorded (Figure 2F,G). GCA^+^ immune cells group showed no significant differences in time with objects (Figure 2G), and the recognition index was significantly lower than that in the PBS group (Figure 2H). To ensure that the observed decrease in recognition index was not due to changes in locomotor activity or exploratory behavior, an open field test (OFT) was conducted before the NORT. In the OFT, both groups of mice exhibited similar levels of total distance traveled (Figure S2D, Supporting Information) and time spent in the center of the field (Figure S2E, Supporting Information).

As Aβ deposition and subsequent nerve damage are widely recognized as the primary pathogenesis of AD,^[^ ^15^ ^]^ we investigated whether the administration of GCA^+^ immune cells could affect Aβ accumulation. Microglia are the primary immune cells in the central nervous system for the clearance of Aβ plaques. We first found that ionized calcium‐binding adapter molecule 1 (IBA1), the marker of microglia that increases upon activation,^[^ ^16^ ^]^ was decreased in the hippocampus and cortex of GCA^+^ immune cells‐transferred mice (Figure 2I; Figure S2F–H, Supporting Information). By contrast, glial fibrillary acidic protein (GFAP), the marker of astrocytes was not affected (Figure S2I, Supporting Information). Consistent with the restrained activation of microglia, GCA^+^ immune cells‐transferred mice revealed a decreased area of Neu‐N immunoreactive cells (Figure 2J,K; Figure S2F,G, Supporting Information) in both the cortex and hippocampus, suggesting that neurons were impaired. Collectively, these results indicated that GCA^+^ immune cells may aggravate the behavioral performance and pathogenesis of Aβ‐treated mice.

### 2.3 Bone Marrow‐Derived GCA^+^ Immune Cells Preferentially Invaded the Brain of AD Mouse Model in a CCR10‐Dependent Manner

**Figure 3:**
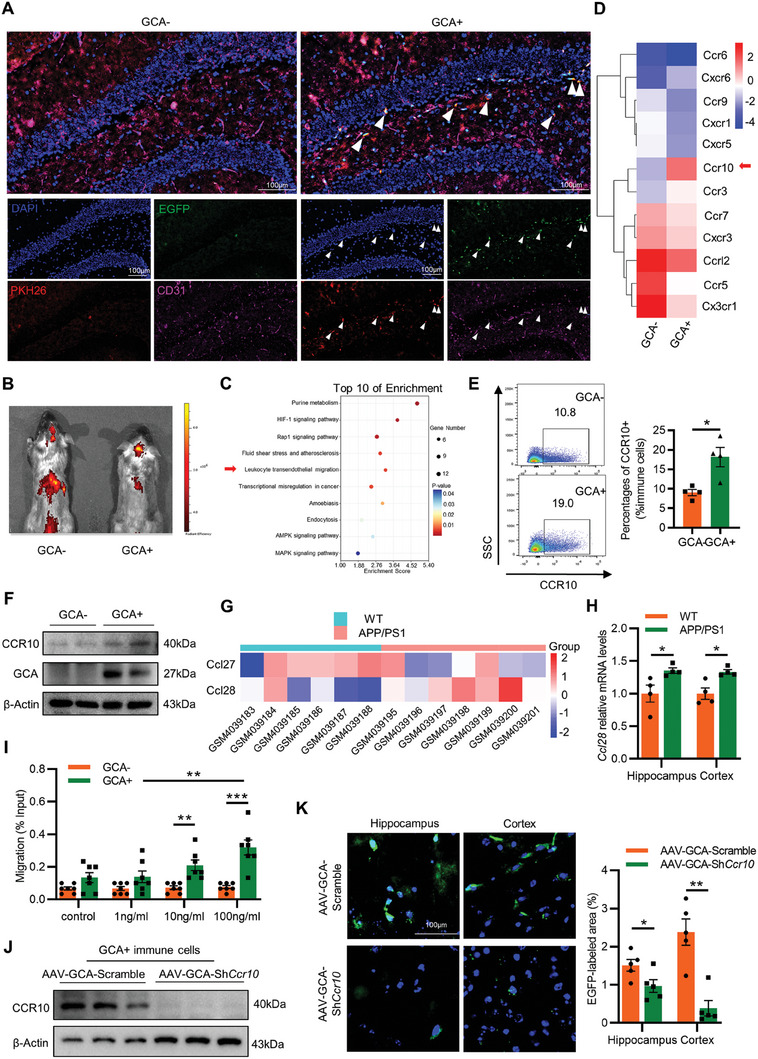
Bone marrow‐derived GCA^+^ immune cells preferentially invaded the brain of AD mouse model in a CCR10‐dependent manner. A) Representative images of PKH26 (red), EGFP (green), CD31 (violet), and nucleus (blue) staining in hippocampus from GCA^+^ immune cells, GCA^−^ immune cells groups. White arrows indicate cells within the brain parenchyma (Scale bar = 100 µm; *n* = 4). B) Representative ex vivo fluorescent images from Aβ‐injected mice treated with DiR‐labeled GCA^+^ immune cells or GCA^−^ immune cells by intravenous injection. C) KEGG enrichment analysis of upregulated genes in GCA^+^ immune cells compared with GCA^−^ immune cells. D) Heatmap of chemokine receptor genes across GCA^+^ immune cells and GCA^−^ immune cells. E) Relative cell proportion of CCR10 in GCA^+^ immune cells and GCA^−^ immune cells (n = 4). (F) Representative immunoblots of CCR10 in GCA^+^ immune cells and GCA^−^ immune cells. G) Heatmap of *Ccl27* and *Ccl28* in brain from APP/PS1 mice and control mice (*Ccl28*, p value < 0.05). H) Relative mRNA levels of *Ccl28* in cortex and hippocampus from APP/PS1 mice and control mice. I) Bar chart of GCA^+^ immune cells and GCA^−^ immune cells migration treated with different concentrations of chemokine, Ccl28 (*n* = 7). J) Representative immunoblots of CCR10 in GCA^+^ immune cells treated with treated with AAV‐CMV‐FLEX‐*S*h*Ccr10* (AAV‐*GCA‐S*h*Ccr10*) or AAV‐CMV‐FLEX‐Scramble (AAV‐*GCA‐* Scramble) (*n* = 3). K) Representative images of GFP (green) and nucleus (blue) staining in cortex and hippocampus from Aβ‐injected AAV‐*GCA‐S*h*Ccr10* mice or AAV‐*GCA‐*Scramble mice. (Scale bar = 100 µm; *n* = 5). Data are shown as the mean ± SEM. For panel (E,H,K): unpaired, two‐tailed Student's t‐test. For panel (I): two‐way ANOVA. ^*^ *p* < 0.05, ^**^ *p* < 0.01, ^***^ *p* < 0.001.

Since GCA^+^ immune cells accelerate AD progression, we next found that weeks after transplantation, PKH26^+^EGFP^+^ immune cells (GCA^+^ immune cells) showed specific infiltration in the brain of GCA^+^ immune cells‐transferred mice, whereas PKH26 signals were rarely detected in the GCA^−^ immune cells‐transferred mice (**Figure** **3**A; Figure S3A, Supporting Information). Consistently, the results of in vivo tracking analysis showed that lipotropic dye DiR‐labeled GCA^+^ immune cells, which were intravenously injected into Aβ‐induced mice, manifested stronger signal accumulation in the brain compared to the GCA^−^ immune cells group (Figure 3B). These results suggested that the GCA^+^ immune cells have a preferential ability to accumulate in the brain.

To elucidate the mechanism underlying the specific chemotaxis of GCA^+^ immune cells, isolated GCA^+^ immune cells and GCA^−^ immune cells were subjected to RNA‐sequencing. KEGG analysis showed that upregulated genes were enriched in HIF‐1 signaling, Rap1 signaling, purine metabolism, and leukocyte trans‐endothelial migration (Figure 3C), all tightly correlated with cell migration. Notably, among those chemokine receptor‐related genes, CCR10, a classical chemokine receptor that facilitates inflammation in the skin^[^ ^17^ ^]^ and rheumatoid arthritis pathogenesis,^[^ ^18^ ^]^ was the most significantly upregulated in GCA^+^ immune cells versus GCA^−^ immune cells (Figure 3D). FACS and western blot analyses verified abundant CCR10 expression in GCA^+^ immune cells (Figure 3E,F). It is reported that the classical ligands of CCR10 are CCL27 and CCL28.^[^ ^19^ ^]^ To figure out whether these chemokines are responsible for recruiting GCA^+^ immune cells to the brain, we re‐analyzed the public database GSE135999 concluding brain data from APP/PS1 mice and control mice. CCL28 was significantly upregulated in the brain of APP/PS1 mice compared with control mice, while CCL27 showed no significant differences (Figure 3G). We further confirmed that the mRNA levels of CCL28 were indeed upregulated in the brain of APP/PS1 mice compared to control mice (Figure 3H). To exactly explore whether the CCL28‐CCR10 axis mediated the chemotaxis effect of GCA^+^ immune cells, we performed a transwell migration assay. GCA^+^ immune cells were sorted by flow cytometry, then seeded in the upper chamber, while CCL28 was added in the lower chamber. After 24 h of coculture, significantly enhanced migration of GCA^+^ immune cells were observed in response to CCL28, but not vehicle. By contrast, GCA^−^ immune cells showed no obvious migration capacity either with or without CCL28 (Figure 3I). To further determine whether CCR10 played an essential role in mediating GCA^+^ immune cell migration in vivo, *Gca‐Cre‐EGFP* mice were injected intramedullary with adeno‐associated virus (AAV)‐delivered short hairpin RNA (ShRNA) targeting *Ccr10* (AAV‐CMV‐FLEX‐*S*h*Ccr10)* or AAV‐CMV‐FLEX‐Scramble, which allows for specific depletion of *Ccr10* in bone marrow GCA^+^ immune cells (Figure 3J). The mice were then injected with Aβ to induce AD progression. Notably, we found that *Ccr10*‐depletion greatly suppressed the infiltration of GCA^+^ immune cells in the hippocampus and cortex (Figure 3K). Together, these data suggested that the CCL28‐CCR10 axis mediates the specific migration of GCA^+^ immune cells to the brain during AD progression.

### 2.4 Intracerebral Injection of Recombinant GCA Protein Exacerbated AD Progression

**Figure 4:**
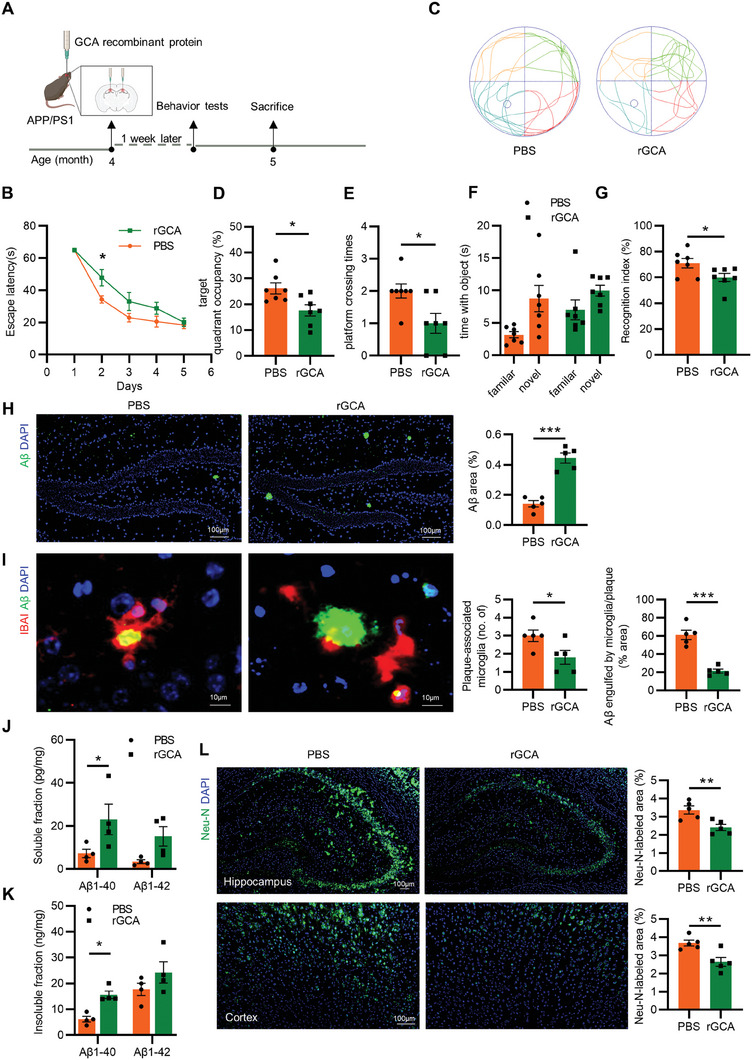
Intracerebral injection of recombinant GCA protein exacerbates AD progression. A) Schematic diagram for recombinant GCA protein (rGCA) treatment. B–E) In the MWM test, B) the escape latency time, C) representative motion trajectories, D) target quadrant occupancy, E) platform crossing times in APP/PS1 mice treated with or without rGCA (*n* = 7). F,G) Time with the object F) and recognition index G) was assessed of the NORT (*n* = 7). H) Representative images and area percentage of Aβ (green) and nucleus (blue) staining in hippocampus. (Scale bar = 100 µm; *n* = 5). I) Representative images of IBA1 (red), Aβ (green), and nucleus (blue) staining, and bar chart of number of plaque‐associated microglia and the volume percentage of Aβ engulfed by microglia in plaque. (Scale bar = 10 µm; *n* = 5). J) ELISA of the concentration of soluble A𝛽_1‐40_ and A𝛽_1‐42_ in APP/PS1 mice treated with or without rGCA (*n* = 4). K) ELISA of the concentration of insoluble A𝛽_1‐40_ and A𝛽_1‐42_ in APP/PS1 mice treated with or without rGCA (*n* = 4). L) Representative images of Neu‐N (green) and nucleus (blue) staining and quantification of Neu‐N labeled area in hippocampus and cortex from APP/PS1 mice treated with or without rGCA. (Scale bar = 100 µm; *n* = 5). Data are shown as the mean ± SEM. For panel (B): two‐way ANOVA. For panel (D,E,G–L): unpaired, two‐tailed Student's t‐test. For panel (F): paired, two‐tailed Student's t‐test. ^*^ *p* < 0.05, ^**^ *p* < 0.01, ^***^ *p* < 0.001.

As GCA^+^ immune cells express abundant GCA (Figure S4A, Supporting Information),^[^ ^11^ ^]^ and also secret GCA in cell culture supernatant (Figure S4B, Supporting Information). We next investigate the direct impact of GCA protein on AD progression. We intracerebrally injected recombinant GCA (rGCA) protein into the hippocampus of 4‐month‐old APP/PS1 mice (**Figure** **4**A). One week later, the behavior performance of APP/PS1 mice was tested. Results showed that behavior performance of rGCA‐treated mice was significantly impaired compared to the control group in terms of escape latency time, target quadrant occupancy, and platform crossing times (Figure 4B–E; Figure S4C, Supporting Information). Both the rGCA and PBS‐treated group of mice showed similar swimming speeds (Figure S4D, Supporting Information). Additionally, the recognition index was significantly reduced in the rGCA group compared to the WT group in the NORT (Figure 4F,G). However, there were no significant differences in locomotor activity and exploratory behavior in the center (Figure S4E,F, Supporting Information) between the rGCA and PBS groups in the OFT.

**Figure 5:**
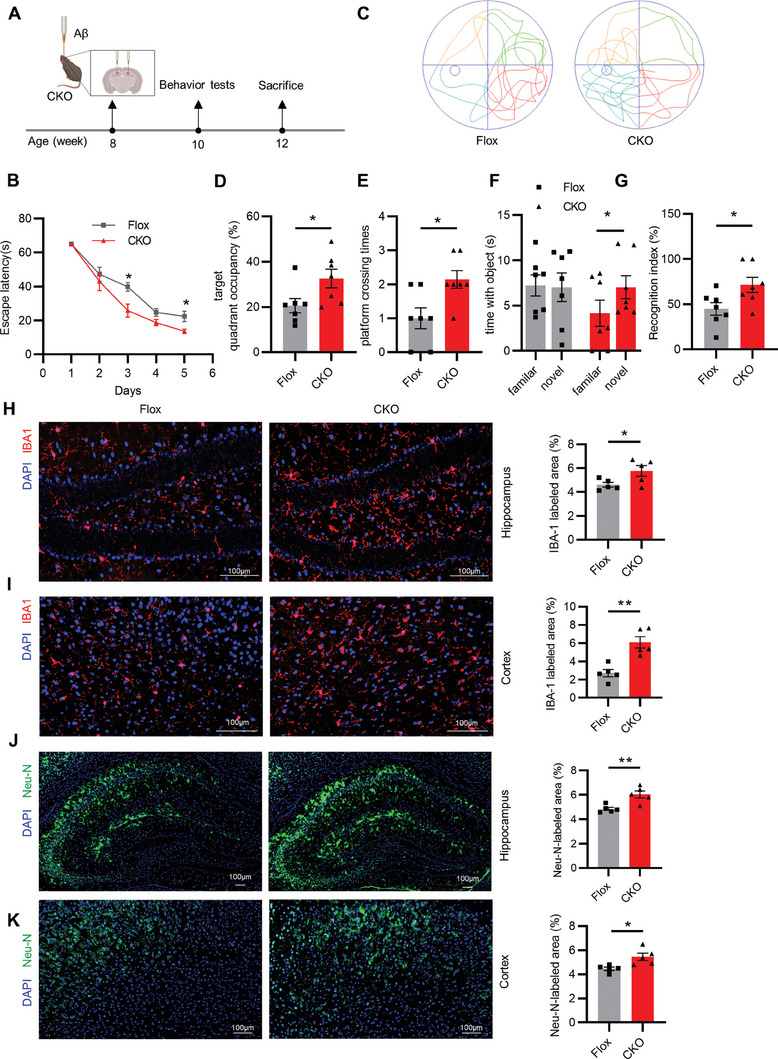
Genetic ablation of GCA in the hematopoietic cells improved cognitive and memory function A) Schematic diagram for treatment and detection time points were indicated. B–E) In the MWM test, B) the escape latency time, C) representative motion trajectories, D) target quadrant occupancy, E) platform crossing times in Aβ‐injected *Gca* hematopoietic knockout mice (*Gca‐Vav1‐CKO; CKO*) and control littermates (*Gca^f/f^*; Flox) (*n* = 7). F,G) Time with the object F) and recognition index G) were assessed of the NOR test (*n* = 7). H) Representative images of IBA1 (red) and nucleus (blue) staining in hippocampus and quantification of IBA1 labeled area from Aβ‐injected CKO mice and Flox mice. (Scale bar = 100 µm; *n* = 5). I) Representative images of IBA1 (red) and nucleus (blue) staining in cortex and quantification of IBA1 labeled area from Aβ‐injected CKO mice and Flox mice. (Scale bar = 100 µm; *n* = 5). J) Representative images of Neu‐N (green) and nucleus (blue) staining in hippocampus and quantification of Neu‐N labeled area from Aβ‐injected CKO mice and Flox mice. (Scale bar = 100 µm; *n* = 5). K) Representative images of Neu‐N (green) and nucleus (blue) staining in cortex and quantification of Neu‐N labeled area from Aβ‐injected CKO mice and Flox mice. (Scale bar = 100 µm; n = 5). Data are shown as the mean ± SEM. For panel (B): two‐way ANOVA. For panel (D,E,G–K): unpaired, two‐tailed Student's t‐test. For panel (F): paired, two‐tailed Student's t‐test. ^*^ *p* < 0.05, ^**^ *p* < 0.01, ^***^ *p* < 0.001.

Furthermore, we evaluated the effect of rGCA on amyloid plaque load in the brain. Our results showed that rGCA‐treated mice had a significantly increased amyloid plaque load in the hippocampus compared to PBS‐treated mice (Figure 4H). Additionally, we also evaluated the number of microglia around the amyloid plaque, which is known to impact plaque composition and toxicity.^[^ ^20^ ^]^ We observed decreased numbers of microglia in the vicinity of an amyloid plaques in the rGCA group, with a lower co‐localization of Aβ and IBA1 compared to the control group (Figure 4I). In line with this, the concentrations of soluble and insoluble Aβ_1‐40_ and Aβ_1‐42_ were increased in the brain of rGCA ‐treated mice at the protein level (Figure 4J,K). In line with the Aβ deposition, rGCA treatment reduced the total area of neurons in hippocampus and cortex (Figure 4L; Figure S4G, Supporting Information). Overall, our results indicated that rGCA treatment can directly exacerbate AD progression.

### 2.5 Genetic Ablation of GCA in the Hematopoietic Cells Improved Cognitive and Memory Function

Based on the above results, we proceeded to investigate whether depletion of *Gca* in immune cells will impact the progression of AD in vivo. Since *Lyz2‐Cre* was reported to be leaky into microglia,^[^ ^21^ ^]^ therefore, we generated hematopoietic cell‐specific depletion mice (*Gca‐Vav1‐CKO*) by crossing *Gca^flox/flox^* and *Vav1‐Cre* mice, which resulted in the deletion of the *Gca* gene in hematopoietic lineage cells.^[^ ^11^ ^]^ We verified that GCA was highly expressed in the bone marrow of *Gca^flox/flox^* but merely expressed in the brain (Figure S5A, Supporting Information). Moreover, GCA was significantly suppressed in the bone marrow of *Gca‐Vav1‐CKO* mice (Figure S5A, Supporting Information). We then induced cognitive impairment in both *Gca‐Vav1‐CKO* and control mice by injecting Aβ into the bilateral hippocampus (**Figure** **5**A). Subsequently, we evaluated the cognitive function of *Gca‐Vav1‐CKO* mice and found that *Gca* depletion exhibited improved behavior performance in the MWM, as evidenced by reduced escape latency time, increased target quadrant occupancy, and platform crossing times compared to control mice (Figure 5B–E; Figure S5B, Supporting Information), while there was no significant difference in the mean swimming speed between the two groups of mice (Figure S5C, Supporting Information). In the NORT, increased time with novel objects and a significant increase in recognition index were observed in *Gca‐Vav1‐CKO* mice compared to control littermates (Figure 5F,G). The OFT results showed no differences in locomotor activity and exploratory behavior between *Gca‐Vav1‐CKO* and control mice, indicating that the increase in recognition index was not due to alterations in these behaviors (Figure S5D,E, Supporting Information).

Consistent with the improved behavior performance, the expression of IBA1 was higher in the hippocampus and cortex of Aβ‐induced *Gca‐Vav1‐CKO* mice (Figure 5H,I; Figure S5F,G, Supporting Information). In line with this, the loss of neurons was greatly alleviated after *Gca* depletion (Figure 5J,K; Figure S5H, Supporting Information). Moreover, neuroinflammation was also suppressed upon *Gca* ablation (Figure S5I, Supporting Information). These results collectively suggested that specific deletion of GCA in hematopoietic cells constrain AD‐related pathological changes and delay the disease progression.

### 2.6 GCA Hampered Amyloid Plaque Clearance via Competitively Binding to LRP1 in Microglia

**Figure 6:**
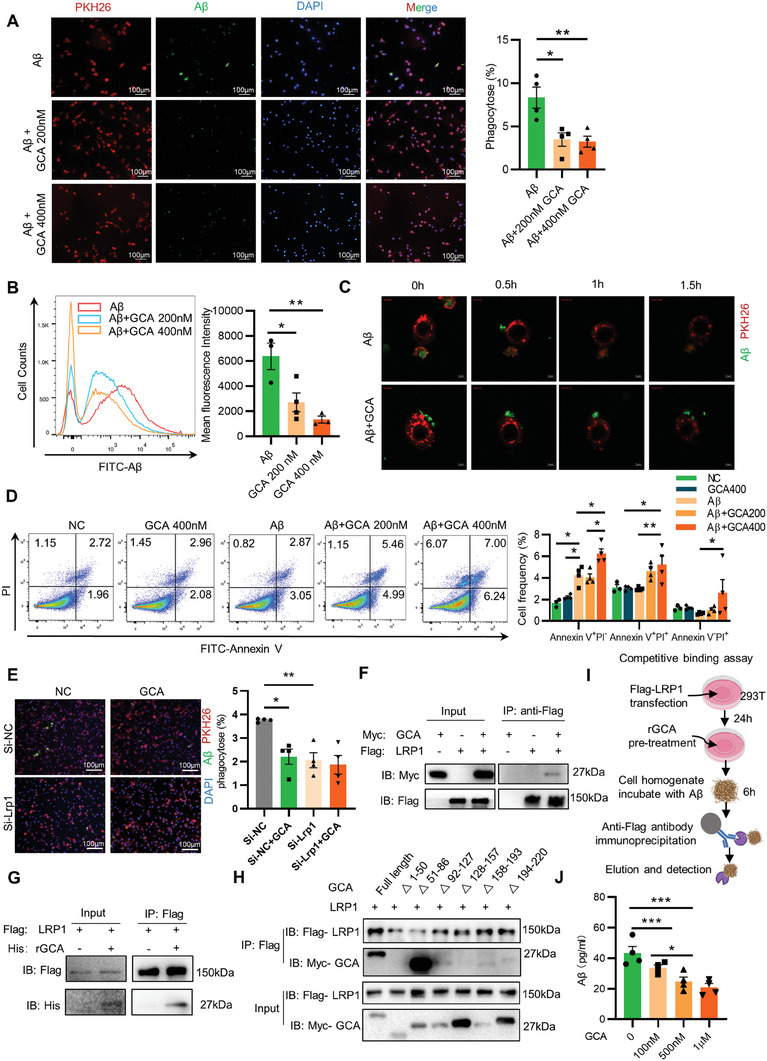
GCA hampered amyloid plaque clearance via competitively binding to LRP1 in microglia. A) Representative images of PKH26 (red), FITC‐Aβ (green), and nucleus (blue) staining in BV‐2 cells after incubating with FITC‐Aβ and different concentration rGCA. (Scale bar = 100 µm; *n* = 4). B) Representative flow cytometry plots of phagocytic FITC‐Aβ in BV‐2 cells (*n* = 3–4). C) Representative live fluorescent images of BV‐2 cells phagocyting FITC‐Aβ at different time points. 30 min after adding FITC‐Aβ was marked as 0 h (Scale bar = 5 µm). D) Representative flow cytometry plots and percentage of different quadrants of SH‐SY5Y treated supernatant from BV‐2 cells after incubating with FITC‐Aβ and different concentration GCA (*n* = 3–4). (E) Representative images of PKH26 (red), FITC‐Aβ (green), and nucleus (blue) staining in LRP1‐knockdown BV‐2 cells or not, then incubating with FITC‐Aβ and 200 nm rGCA. (Scale bar = 100 µm; *n* = 4). F) Co‐immunoprecipitation (Co‐IP) analysis of Flag‐LRP1 and Myc‐GCA. G) IP analysis of GCA and LRP1 binding in HEK 293T cells overexpressed with LRP1 and treated with or without rGCA. H) IP analysis of various GCA deletion mutants and their binding to LRP1. I) Flow chart about pull down Aβ binding to LRP1. J) ELISA of the concentration of Aβ binding to LRP1 treated with different concentration of rGCA (*n* = 4). Data are shown as the mean ± SEM. For panel (A), (B), (E) and (J): one‐way ANOVA. For panel (D): two‐way ANOVA. ^*^, *p* < 0.05, ^**^, *p* < 0.01, ^***^, *p* < 0.001.

We next set out to delineate the mechanisms of GCA on Aβ deposition. *Gca* depletion in hematopoietic cells had no significant effects on the levels of APP generation or Aβ‐degrading enzymes (Figure S6A–D, Supporting Information). Microglia are the main glial cells that participate in Aβ clearance.^[^ ^22^ ^]^ Amyloid clearance is affected by several factors, especially activation of cellular uptake.^[^ ^23^ ^]^ To determine whether GCA affect Aβ clearance, we treated BV‐2 microglia cells, which facilitates clearance of Aβ from extracellular space,^[^ ^24^ ^]^ with different concentrations of recombinant GCA protein (rGCA).^[^ ^11^ ^]^ After rGCA incubation, FITC‐Aβ peptides (green) were added to BV‐2 cells for another 2 h. Immunofluorescent staining showed that rGCA treatment markedly suppressed the uptake of FITC‐Aβ in BV‐2 cells (**Figure** **6**A). FACS‐based internalization assay also showed that rGCA treatment diminished the percentage of Aβ‐phagocytic microglia in a dose‐dependent manner (Figure 6B). Additionally, to address whether Aβ was stuck on cell surface or taken up by the cells, we extracted membrane and cytosol protein from FITC‐Aβ treated BV‐2 cells and detected their fluorescence intensity, respectively. Data showed that the fluorescence intensity of cytosol protein was much stronger than that from the membrane protein (Figure S7A, Supporting Information). Permeabilization of cells would also help to distinguish the levels of surface‐bound and cytosol Aβ. Flow cytometry results showed that Aβ^+^ cells were slightly detected in non‐permeabilized BV2 cells, where its proportions markedly increased in permeabilized cells (Figure S7B, Supporting Information). These results suggest that the majority of Aβ was up taken by the cells but not stuck on cell surface. To be note, rGCA treatment reduced the proportions of Aβ^+^ BV‐2 cells under both staining conditions (Figure S7B, Supporting Information). Consistently, live cell imaging dynamically exhibited that rGCA blocked Aβ phygocytosis in BV‐2 cells (Figure 6C). We suspected more Aβ remaining in the supernatant from rGCA treatment group as extracellular residual Aβ had neurotoxicity^[^ ^15^ ^]^ and led to neurotoxic inflammatory cytokine secretion.^[^ ^24^ ^]^ We treated human neuroblastoma cell line (SH‐SY5Y) with the above supernatant from BV‐2 cells incubated with or without rGCA and Aβ. Notably, supernatant from BV‐2 cells treated by rGCA caused more severe apoptosis of SH‐SY5Y cells (Figure 6D). These results demonstrate a critical role of GCA in blocking Aβ clearance by microglia.

We next set out to identify the receptor that mediates the effect of GCA. Notably, our previous study predicted low‐density lipoprotein receptor‐related protein 1 (LRP1), a ubiquitously expressed transport receptor in microglia and vasculature, was one of the potential receptors for GCA in bone marrow derived mesenchymal stem cells.^[^ ^11^ ^]^ To address whether GCA blocked Aβ clearance though LRP1, we knocked down *Lrp1* in BV‐2 cells with *Lrp1* siRNA. Compared to the scramble control cells, the phagocytosis of Aβ was significantly inhibited in *Lrp1*‐deficient cells. Further, though rGCA treatment could block Aβ phagocytosis, the inhibition of Aβ phagocytosis by rGCA treatment was not further reduced after *Lrp1* ablation (Figure 6E). To verify the direct binding of GCA to LRP1 in vitro, Myc‐tagged GCA and Flag‐tagged LRP1 were transfected into human embryonic kidney (HEK) 293T cells via plasmids transfection. And the HEK 293T cells were collected to conduct immunoprecipitation with by anti‐Flag antibody. The results of immunoblots showed a strong band of Myc staining in the Flag immunoprecipitants, which indicated the interaction of GCA and LRP1 (Figure 6F). We further confirm that rGCA binds to the extracellular domain of LRP1 as recombinant rGCA was precipitated in the HEK 293T cells that transfected with LRP1 after coculture (Figure 6G). To further depict the function domain of GCA that specifically interacts with LRP1, we constructed 6 mutants of GCA according to different domain positions. Notably, nucleotides 1–50 depletion mutants (GCA^△1‐50^) and 128–157 depletion mutants (GCA^△128‐157^) of GCA no longer interact with LRP1 (Figure 6H), indicating that the two domains are responsible for the bind of GCA to LRP1.

To clarify whether the combination of GCA with LRP1 affected the function of LRP1 in Aβ clearance, the competition binding assay was performed. Flag‐tagged LRP1‐overexpressed cells were pre‐incubated with different concentrations of rGCA overnight. Cell lysates were collected and mixed with Aβ peptides for indicated times. Pull‐down Aβ using anti‐flag antibody‐coated agarose beads suggest that GCA pre‐incubation competitively abolished the binding of LRP1 with Aβ peptides in a dose‐dependent manner (Figure 6I,J). Together, these results suggest that GCA competitively binds to LRP1 to inhibit Aβ endocytosis in microglia.

### 2.7 GCA‐Neutralizing Antibody Treatment Alleviated AD Progression

**Figure 7:**
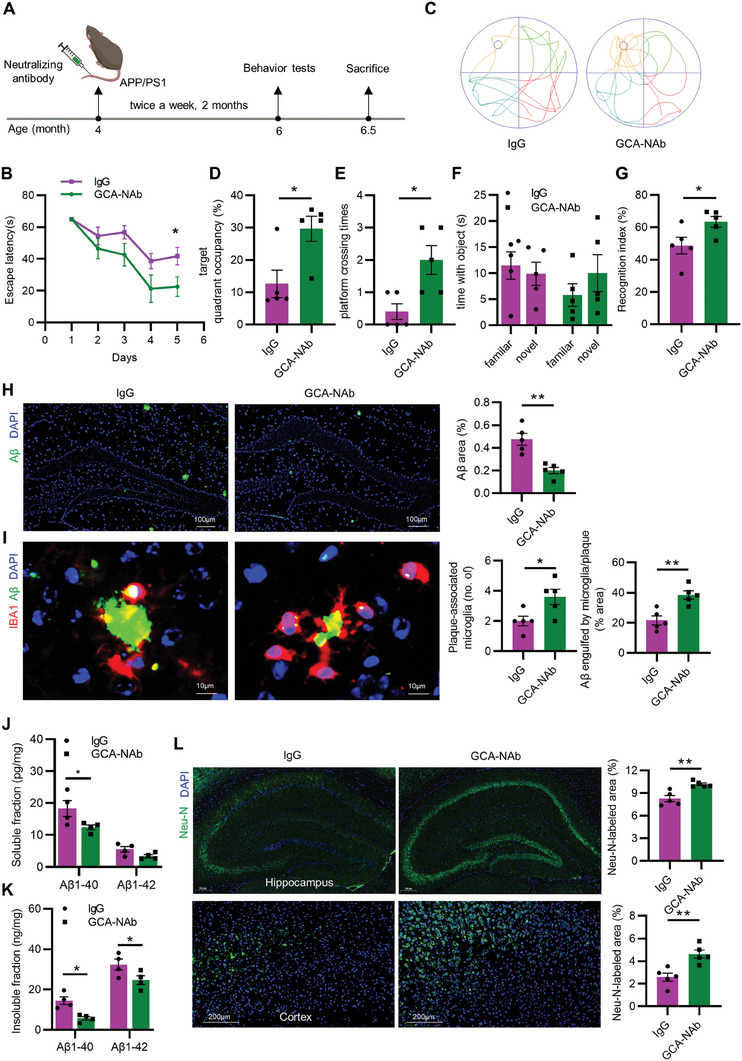
GCA‐Neutralizing antibody treatment alleviated AD progression. A) Schematic diagram for treatment and detection time points were indicated. B–E) In the MWM test, B) the escape latency time, C) representative motion trajectories, D) target quadrant occupancy, E) platform crossing times in APP/PS1 mice treated with GCA neutralizing antibody (GCA‐NAb) or IgG control (*n* = 5). F,G) Time with the object F) and recognition index G) were assessed of the NORT (*n* = 5). H) Representative images and area percentage of Aβ (green) and nucleus (blue) staining in hippocampus from APP/PS1 mice treated with GCA‐NAb or IgG control. (Scale bar = 100 µm; *n* = 5). I) Representative images of IBA1 (red), Aβ (green), and nucleus (blue) staining, and bar chart of number of plaque‐associated microglia and the volume percentage of Aβ engulfed by microglia in plaque. (Scale bar = 10 µm; *n* = 5). J) ELISA of the concentration of soluble A𝛽_1‐40_ and A𝛽_1‐42_ in APP/PS1 mice treated with GCA‐NAb or IgG control (*n* = 4). K) ELISA of the concentration of insoluble A𝛽_1‐40_ and A𝛽_1‐42_ in APP/PS1 mice treated with GCA‐NAb or IgG control (*n* = 4). L) Representative images of Neu‐N (green) and nucleus (blue) staining and quantification of Neu‐N labeled area in hippocampus and cortex. (Scale bar = 200 µm; n = 5). Data are shown as the mean ± SEM. For panel (B): two‐way ANOVA. For panel (D,E,G–L): unpaired, two‐tailed Student's t‐test. For panel (F): paired, two‐tailed Student's t‐test. ^*^ *p* < 0.05, ^**^ *p* < 0.01.

Our previous research has demonstrated a critical role of GCA neutralizing antibody in delaying age‐related bone loss.^[^ ^11^ ^]^ Here, we investigated whether GCA neutralizing antibody could restrain the AD progression. Early‐stage AD mouse model (4‐month‐old) were treated with GCA neutralizing antibody via tail vein injection (1 mg kg^−1^, twice a week) for two months, while the control group received an equal amount of IgG (**Figure** **7**A). Behavioral tests were conducted when the mice reached 6‐month‐old (late‐stage AD). Compared to the control group, the mice treated with GCA neutralizing antibody had a significantly reduced escape latency, increased target quadrant occupancy and platform crossing times (Figure 7B–E; Figure S8A, Supporting Information). The time spent with novel objects and the recognition index was also significantly increased in the GCA neutralizing antibody group compared to the control group (Figure 7F,G). There was no significant difference between the mice treated with GCA neutralizing antibody and the control group in mean swimming speed (Figure S8B, Supporting Information), total distance traveled (Figure S8C, Supporting Information) and time spent in the center (Figure S8D, Supporting Information).

Moreover, the amyloid plaque load in the hippocampus of mice treated with GCA‐neutralizing antibody was significantly reduced (Figure 7H). Compared to the mice treated with IgG, mice treated with GCA neutralizing antibody showed more plaque‐associated microglia and as well as a higher ratio of Aβ engulfed by microglia co‐localization level in the brains (Figure 7I; Figure S8E, Supporting Information). In line with this, the concentration of soluble or insoluble Aβ_1‐40_ and Aβ_1‐42_ in the brain tissue of mice treated with GCA‐neutralizing antibodies were lower than those in the control group (Figure 7J,K). The neuron loss in the hippocampus and cortex was also dramatically alleviated (Figure 7L; Figure S8F, Supporting Information). Consistently with the attenuated neuropathology, neuroinflammation was also suppressed after GCA neutralizing antibodies treatment (Figure S8G, Supporting Information). Taken together, our findings suggest that GCA neutralizing antibody treatment could delay the progression of AD and may become a potential novel therapeutic option for AD.

## 3 Discussion

**Figure 8:**
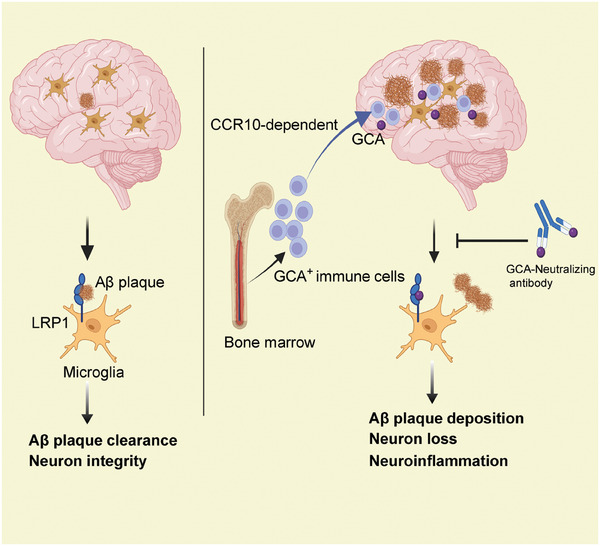
Schematic diagram. Bone marrow‐derived GCA^+^ immune cells migrate to the mouse brain through the CCR10‐CCL28 axis and contribute to the disease progression. GCA, which mediates the effect of GCA^+^ immune cells, competitively binds to the receptor LRP1 on microglia and impairs their ability to clear Aβ, leading to neuronal loss and neuroinflammation. Neutralizing antibody targeting GCA (GCA‐NAb) can significantly prevent this pathological process and improves the cognitive and memory function.

In this study, we have revealed bone marrow‐derived GCA^+^ immune cells that migrate to the mouse brain through the CCR10‐CCL28 axis and contribute to the progression of AD. These cells secrete abundant GCA, which competitively binds to the receptor LRP1 on microglia and impairs their ability to phagocytose and clear Aβ plaque load. Additionally, we identified a neutralizing antibody targeting GCA that significantly improves the cognitive and memory function in mice (**Figure** **8**). Our study provides novel insights into the involvement of bone marrow‐derived immune cells in the pathogenesis of AD.

AD is an age‐related neurodegenerative disorder. With aging, the immune system also undergoes remarkable changes that collectively are known as immunosenescence, which not only leads to impaired immunity but also drives aging and damage in non‐immune organs such as the brain.^[^ ^25^ ^]^ Studies have shown that immune system aging is closely related to the onset and progression of AD. Older adults have a significantly higher capacity for monocyte infiltration into brain tissue than young individuals, and these monocytes from older adults are more capable of inducing pro‐inflammatory responses, thereby promoting brain aging.^[^ ^26^ ^]^ Moreover, inhibition of the prostaglandin E2 signaling pathway in peripheral macrophages improves their hippocampal synaptic plasticity and spatial memory.^[^ ^8^ ^]^ These studies support the notion that aging of the peripheral immune system may directly drive brain aging, leading to central neurodegenerative diseases such as AD. GCA^+^ immune cells, a group of myeloid cells with pro‐inflammatory and pro‐aging properties, accumulate in the bone marrow of aged mice and induce bone aging.^[^ ^11^ ^]^ Here, we found that GCA was highly expressed in the peripheral macrophages of AD patients and mice, and transplantation of GCA^+^ immune cells or intracerebral injection of recombinant GCA protein directly aggravated cognitive and memory impairments, whereas deletion of the *Gca* gene in the hematopoietic cells improved cognitive and memory function. This discovery undoubtedly represents a significant addition to previous research and further supports the hypothesis that the peripheral immune system may influence neurodegeneration.

Chemokines and their receptors play a vital role in regulating the directional migration of immune cells between the circulatory system and organs.^[^ ^27^ ^]^ For instance, in response to radiation or cerebral ischemia, microglia secrete chemokines such as CCL2 and CCL8 to attract peripheral CCR2^+^/CCR5^+^ CD8^+^T cells to infiltrate the brain, triggering secondary brain damage.^[^ ^28^ ^]^ In AD, the accumulation of Aβ and tau leads to glial cell activation and blood‐brain barrier damage, and activated microglia and astrocytes persistently release cytokines and chemokines to further impair the blood‐brain barrier.^[^ ^29^ ^]^ Peripheral immune cells can be recruited to diseased brain tissues through these factors and play important regulatory roles. In our study, we conducted RNA sequencing on GCA^+^ and GCA^−^ immune cells to investigate the mechanism by which GCA^+^ immune cells migrate to the brain. We found that GCA^+^ immune cells specifically expressed high levels of CCR10, a receptor for CCL28, which is involved in immune and inflammatory regulation in various diseases, such as tumors,^[^ ^30^ ^]^ spinal cord injury,^[^ ^31^ ^]^ and skin diseases.^[^ ^32^ ^]^ However, there are currently no studies confirming the role of the CCR10‐CCL28 axis in AD. We confirmed the elevation of CCL28 in the cortex of AD mice through array datasets analysis and experiments. By specifically knocking down of *Ccr10* in bone marrow GCA^+^ immune cells, we demonstrated that the CCL28‐CCR10 axis recruited peripheral GCA^+^ immune cells invading the damaged blood‐brain barrier into the brain, further accelerating the progression of AD. Our study identifies the CCR10‐CCL28 axis as a critical pathway for the migration of peripheral immune cells into the brain, participating in the vicious circle that accelerates AD progression.

In this study, we confirmed the binding of GCA to LRP1 and that treatment with rGCA resulted in a concentration‐dependent decrease in Aβ phagocytosis by BV‐2 cells. Consistent with previous reports that competitive binding of Aβ to LRP1 by APOE on astrocytes may interfere with AD severity,^[^ ^33^ ^]^ we have shown that increased binding of GCA to LRP1 led to decreased binding of Aβ to LRP1 in microglia, accompanied by increased Aβ deposition. LRP1 has been extensively studied in AD as it participates in the clearance of Aβ by various cell types, including microglia,^[^ ^34^ ^]^ neurons,^[^ ^35^ ^]^ astrocytes,^[^ ^36^ ^]^ endothelial cells,^[^ ^37^ ^]^ vascular smooth muscle cells,^[^ ^38^ ^]^ oligodendrocytes,^[^ ^39^ ^]^ and pericytes.^[^ ^40^ ^]^ Moreover, studies suggest that microglia remove plaque associated Aβ while the majority of monomeric Aβ is cleared through receptor‐mediated transcytosis across the brain‐blood barrier.^[^ ^41^ ^]^ Therefore, it is possible that GCA may also impair the function of other cell types to clear brain Aβ,^[^ ^42^ ^]^ which collectively elevated brain Aβ levels and aggravated spatial learning and memory deficits. The liver physiologically clears blood Aβ and regulates brain Aβ levels by LRP1.^[^ ^43^ ^]^ There also exists a possibility that GCA would function on hepatocytes to ablate peripheral Aβ clearance. Additionally, inflammation also accelerates AD progression.^[^ ^44^ ^]^ We previously showed that GCA is a proinflammatory cytokine highly increased during aging.^[^ ^11^ ^]^ Thus, GCA may also function to instigate brain inflammation.

Existing drugs and cognitive behavioral therapies only help to alleviate symptoms of AD and lack a curable or effective drugs to slow down disease progression.^[^ ^45^ ^]^ Immune therapy targeting AD has been a hot topic in recent years. In ongoing clinical trials of AD‐related immune therapy, whether active or passive, mainly target Aβ monomers. However, the results of these clinical trials have not shown significant clinical benefits in terms of slowing down cognitive decline, or have faced controversies and safety issues.^[^ ^46^ ^]^ The immune system and the central nervous system have complex interconnections, and once this balance is disrupted, it can lead to neuroinflammation and neurodegenerative diseases.^[^ ^47^ ^]^ Therefore, targeting peripheral immune cells represents a new direction in AD treatment. Peripheral administration of PD‐1 specific antibody can promote myeloid cell (CD45^high^Cd11b^+^) recruitment to the CNS and improves memory in 5XFAD and APP/PS1 mice.^[^ ^48^ ^]^ The use of neutralizing antibodies can effectively prevent T cells from entering the brain parenchyma, and improve brain atrophy, neurofibrillary tangle pathology, microglial activation, and learning and memory function in a Tau AD mouse model.^[^ ^49^ ^]^ Consistent with the study discussed above, treatment with the GCA neutralizing antibody at the early stages of AD (4‐month APP/PS1 mice) accelerates Aβ clearance and delays AD progression. Our GCA‐neutralizing antibody represents an important advancement in this field of research, opening up new avenues for preventing AD progression.

Taken together, our findings demonstrate that peripheral GCA^+^ immune cells preferentially migrate to the brain and accelerate the progression of AD, which provides novel evidence for AD progression and holds significant implications for the constrain of neurodegenerative diseases.

## 4 Experimental Section

### Animal

The study obtained *Gca‐Cre‐EGFP* mice, *Gca^flox/flox^* mice, and *Vav1‐Cre* mice from Cyagen (China). 8‐week‐old young C57 mice were purchased from Hunan SJA Laboratory Animal Company in China. Four‐month‐old APP/PS1 mice were purchased from Cyagen (China). APP/PS1 carries two transgenes associated with AD mutations in Alzheimer's disease: chimeric mouse/human amyloid precursor protein (Mo /HuAPP695swe) and mutated human PSEN1 (PS1‐dE9), both of which target CNS neurons under the control of mouse Prion promoters. The WT mice used as control of APP/PS1 mice were their littermates. Amyloid plaques begin to appear in the cortex at ≈4 months of age and in the hippocampus at ≈6 months. All mice were kept in the specific pathogen‐free facility of the Laboratory Animal Research Center of Central South University at 22–24 °C with a 12 h dark/light cycle, and with ad libitum access to a regular chow diet and water. Animal experiments were approved by the Animal Ethics Committee (202109049) and followed the Guidelines for the Care and Use of Laboratory Animals at Xiangya Hospital of Central South University.

### Study Population

In this study, a total of 20 cognitive normal individuals and 20 patients diagnosed with AD were recruited (Table S1, Supporting Information). The AD patients were not familial but sporadic cases. The AD diagnoses were confirmed by at least two experienced doctors from Xiangya Hospital according to the National Institute of Aging and Alzheimer's Association (NIA‐AA) criteria. Cognitive normal individuals were recruited from the physical examination center of Xiangya Hospital. Individuals with a Mini‐Mental State Examination (MMSE) score exceeding or equal to 27 were included as cognitively normal, following previously established standards.^[^ ^50^ ^]^ The study was approved by the Committee of Clinical Ethics at Xiangya Hospital of Central South University (2023030141). Informed consent was obtained for humans after the nature and possible consequences of the studies were explained.

### Brain Stereotaxic Injection

The *Gca‐Cre‐EGFP* mice, or C57 mice, or *Gca^flox/flox^;Vav1^cre^* mice and control mice at 8–10 weeks of age were anesthetized and placed in a stereotaxic frame, a skin incision was made, and holes were drilled at x (±2.0 mm from bregma) and y (−2.0 mm from bregma). 3 µL A𝛽40 (1 µg µL^−1^) or solvent was bilaterally administered at z‐depths of 2.0 mm into the hippocampus.^[^ ^14b^ ^]^ The A𝛽1‐40 monomer solution (ChinaPeptides, Shanghai, China; MedChemExpress, HY‐P0265A) was incubated at 37 °C for 48–72 h before use. For APP/PS1 mice, at 4 months of age, were injected total of 5 µg of GCA protein (MedChemExpress, HY‐P70334) in 5 µL of PBS, or 5 µL of PBS was delivered to bilaterally hemispheres. The syringe was left in place for 10 min after each injection before being withdrawn slowly. Behavior tests were performed at indicated time.

### Morris Water Maze (MWM)

The apparatus consisted of a white circular pool (120 cm diameter) filled with water at 22–24 °C and divided into four quadrants. An invisible circular platform was placed in the middle of one of the quadrants and submerged 1 cm below the surface. A commercial animal video‐tracking analysis system (AVTAS ver4.0; Wuhan, China) was used for the MWM. Animals were first subjected to water maze training and four trials from four different fixed start positions each day. Each trial lasted 65 s or until the animal found the platform. All animals were left on the platform for 10 s (including those who failed to locate it). After the last training session, the mice were subjected to the probe trail for 65 s. The platform was removed from the pool in the probe trial, and the time spent in the platform quadrant and the platform crossing time were measured.

### Open Field Test (OFT)

Mice were carried to the behavior room to habituate at least 30 min before starting the test. Mice were then placed into the open field arena (made of opaque white plastic material, 50 cm × 50 × 40 cm) and allowed to explore the arena for 10 min. Total distance (cm) and percentage of time spent in the center (22 cm × 22 cm) were quantified.

### Novel Object Recognition Test (NORT)

The experimental apparatus used in this study was the same square box made of opaque white plastic (50 cm×50 cm) used in the open field test. The mice were first habituated to the apparatus for 10 min, and the test served as OFT. After 24 h, two identical objects were positioned in two corners and 5 cm away from each adjacent arena wall. Then the mice were placed in the arena and allowed to explore the arena and objects for 10 min. After 1 h, the mice were placed in the same box, but one of objects were replaced by another object with different color and shape. The time spent exploring the familiar and novel object was also measured for 3 min. The recognition index was calculated as the exploration time of the objects in the novel object/total object exploration time.

### Single‐Cell RNA Sequencing (scRNA‐Seq) And Bioinformatics Analysis

In this study, the scRNA‐seq dataset (GSE181279) was obtained from the GEO database and analyzed using Seurat v4 in R software.^[^ ^51^ ^]^ The dataset was carefully pre‐processed to exclude cells with transcript counts below 100 or above 8000, as well as potential empty droplets, low‐quality cells, and multiplets. Furthermore, cells displaying transcript abundance greater than 10% derived from mitochondrial genes were also excluded from downstream analysis. Gene expression measurements for each cell were standardized and log‐transformed using the “LogNormalize” approach. A Seurat workflow was then implemented to identify the 2000 most highly variable genes in each replicate through variance stabilizing transformation (“vst”). Additionally, 2000 integration anchors were established to avoid results that are excessively specific to certain datasets. These anchors served as an input in the dataset integration procedure. Finally, 2D UMAP was utilized to visualize the clustering outcomes.^[^ ^52^ ^]^

### Bone Marrow Monocytes/Macrophage Isolation

Bone marrow cells were obtained from *Gca‐Cre‐EGFP* mice. Cells were co‐stained with BV510‐CD11b, PE‐Cy7‐Ly6C, PE‐Ly6G (Biolegend, San Diego, USA). Both GCA^+^ immune cells (CD11b^+^Ly6G^−^Ly6C^+^EGFP^+^) and GCA^−^ immune cells (CD11b^+^Ly6G^−^Ly6C^+^EGFP^−^) from bone marrow cells of *Gca‐Cre‐EGFP* mice were sorted by FACS Aria (BD Biosciences). 2 × 10^6^ isolated cells were labeled by PKH26 according to the manufacturer's instructions. Then, labeled cells were transferred into Aβ‐induced WT mice intravenously.

### GCA Neutralizing Antibody Treatment

GCA‐NAb (SinoBiological, 22ZN27C, 1 mg k^−1^g) or IgG control injection by tail intravenous (i.v.) injection twice a week for 2 months.

### Bioinformatics Analysis of RNA‐Seq

GCA^+^ immune cells (CD11b^+^Ly6G^−^Ly6C^+^EGFP^+^) and GCA^−^ immune cells (CD11b^+^Ly6G^−^Ly6C^+^EGFP^−^) were sorted for further RNA‐Seq conducted by OE Biotech Co., Ltd (Shanghai, China). Differentially expressed genes were identified on the basis of *p* < 0.05 and Fold change ≥ 2.0. KEGG displaying the function of differentially upregulated gene was generated by OE cloud (https://cloud.oebiotech.com). Heatmap disclosed the expression of chemokine receptors between these two cell types was generated by OE cloud (https://cloud.oebiotech.com). Ccl27a and Ccl28 expression were download from APP/PS1 and WT cortex samples in GSE135999, and then was presented as a heatmap by OE cloud (https://cloud.oebiotech.com).

### Immunofluorescence Staining

Fresh brain tissues were isolated, post‐fixed and then embedded in OCT for cryosection. Brain tissue slices with the thickness of 10 µm were cut by a microtome, and blocked with in blocking buffer (0.03% Triton‐X 100, 3% BSA in PBS), then, treated with primary antibodies as following, GCA (PA5‐77127, 1:200, Invitrogen), Neu‐N (MAB377,1:100, MD Millipore), IBA1 (ab178846, 1:200, Abcam), F4/80 (ab6640, 1:200, Abcam), Aβ (15126S, 1:200, Cell Signaling), CD31 (Servicebio, GB12063). Subsequently, the brain tissue slides were incubated with Alexa Fluor 488 conjugated anti‐Mouse (Invitrogen, A21202, 1:200) or Alexa Fluor 488 conjugated anti‐Rabbit (Invitrogen, A21206, 1:200) or Alexa Fluor 555 conjugated anti‐Rabbit (Invitrogen, A31572, 1:200) or Alexa Fluor 555 conjugated anti‐Rat (Invitrogen, A‐21434, 1:200) or Alexa Fluor 647 conjugated anti‐mouse (Invitrogen, A‐21235, 1:200) secondary antibodies. Nuclei were counter‐stained with DAPI (Sigma). All the fluorescence pictures were captured by a fluorescence microscope.

### Uptake of Aβ40 in the Presence of Recombinant GCA Protein in BV‐2 Cells

BV‐2 cells were cultured in MEM (NEAA) and supplemented with 10% FBS and 1% PS at 37 °C and 5% CO_2_ in humid atmosphere. After PKH26 labeling, BV‐2 cells were seeded in 24‐well plate. Different concentration of rGCA was pre‐treated BV‐2 cells for 24 h. Subsequently, 5 µg mL^−1^ FITC‐Aβ40 (ChinaPeptides, Shanghai, China) was added in culture medium for another 2 h. Then cells were washed by PBS and stained by DAPI, then visualized with a Microscope, or harvested and subjected to the FACS analysis. To address whether Aβ was stuck on cell surface or taken up by the cells, after adding Aβ40 (ChinaPeptides, Shanghai, China) in BV2‐cells for 2 h, cells were collected and treated with or without fixation/permeabilization (BD Biosciences, San Jose, USA) for 30 min at 4 °C. Then cells were incubated with Aβ antibody (sc‐28365, 1:100, Santa Cruz Biotechnology) for 1 h and Alexa Fluor 488 conjugated anti‐mouse (Invitrogen, A21202, 1:200) for 30 min at 4 °C. Data were analyzed by FlowJo V10 (BD Biosciences, San Jose, USA).

### Effect of Supernatant from Aβ40 Treated BV‐2 Cells on the Apoptosis of SH‐SY5Y Cells

SH‐SY5Y cells were cultured in MEM/F12 and supplemented with 10% FBS and 1% PS at 37 °C and 5% CO_2_ in humid atmosphere. SH‐SY5Y cells were seeded in 24‐well plate. Supernatant from Aβ40 treated BV‐2 cells with different concentration of GCA was replaced culture medium of SH‐SY5Y cells for 48 h. After incubation time, cells were harvest and stained with Annexin V/PI, then analysis under FACS.

### ELISA Analyses

Levels of Aβ_1–40_ and Aβ_1–42_ in mouse hippocampal tissue extractions were measured as described before,^[^ ^53^ ^]^ according to the manufacturer's instructions (Elabscience, Wuhan, China). For Aβ binding, LRP1‐overexpressed cells were pretreated with rGCA for 24 h, then cell lysate was collected and mixed with Aβ. After 6 h incubating, ProteinA/G magnetic beads pretreated with Flag (14793S, 1:100, Cell Signaling Technology) were added to the mixture and incubated further with indicated time. Then, protein was eluted from magnetic beads by 0.1 m glycine (pH 2.5). Elution was added 1 m Tris (pH 7.5) for neutralization to maintain biological activity, then detect Aβ levels by ELISA.

The concentration of GCA in serum was measured using ELISA kits from MYBioSource (MBS7607211). Al procedures were performed according to the manufacturer's instructions.

### Intramedullary Injection of Adeno‐Associated Virus

Recombinant adeno‐associated serotype 9 viruses with CMV promoter for *Ccr10* knockdown in GCA^+^ immune cells (AAV‐CMV‐FLEX‐*S*h*Ccr10*) or scramble control AAV‐CMV‐FLEX‐Scramble were purchased from GeneChem Company (Shanghai, China) and injected intramedullary into the 8‐week‐old *Gca‐Cre‐EGFP* mice.

### Flow Cytometry

For brain cells staining, the *Gca‐Cre‐EGFP* mice were first injected with 2 ug of phycoerythrin (PE)–conjugated anti‐CD45 antibody as previously described.^[^ ^54^ ^]^ After 10 min, mice were euthanized. Brains were infused, minced and digested for 30 min with 1.5 mg mL^−1^ collagenase IV and 0.2 mg mL^−1^ DNase I at 37 °C. After centrifugation and washing, the pellet was first incubated with an anti‐Fc receptor (Biolegend, San Diego, CA) to reduce nonspecific binding of antibodies, followed by incubation with the indicated antibodies for 20–30 min at 4 °C. Then, cells were stained with PE‐CY7‐CD45 and BV510‐CD11b (Biolegend, San Diego, USA) for 30 min at 4 °C. For CCR10 staining, obtained bone marrow cells were stained with the CCR10 antibody (Proteintech, Rosemont, USA) for 1 h at 4 °C. The cells were further incubated with Alexa fluor 647‐anti rabbit IgG (Invitrogen, Carlsbad, USA) and APC‐Cy7‐CD45, BV510‐CD11b, PE‐Cy7‐Ly6C, PE‐Ly6G (Biolegend, San Diego, USA) for 30 min at 4 °C. All antibodies were diluted according to the manual from the manufacturer’ s website. Stained cells were collected by BD FACSCanto II system and analyzed by FlowJo V10. Dead cells and doublets were removed by FSC‐A/FSC‐H gating. Data were analyzed by FlowJo V10 (BD Biosciences, San Jose, USA).

### Immunoprecipitation and Western Blot Analysis

Overexpression of Flag‐LRP1 and Myc‐GCA in HEK 293 T cells was conducted according to the manufacturer's instructions (INVI DNA RNA Transfection Reagent, Invigentech, USA). Considering the full‐length LRP1 was too large to easily and successfully construct plasmid and manipulation, thus a functional mini‐receptor of LRP1 (mLPR4) was used in our study, referring to the functional fragments in Nature.^[^ ^55^ ^]^ Further experiments were performed 48 h after transfection, as previously did.^[^ ^56^ ^]^ Briefly, total cell lysates were centrifuged and the supernatant was collected and incubated with ProteinA/G magnetic beads pretreated with Flag (14793S, 1:100, Cell Signaling Technology) overnight at 4 °C. Immuno‐precipitates were separated by SDS–PAGE and blotted onto a polyvinylidene difluoride (Bio‐Rad Laboratories) membrane. The membrane was incubated with antibodies against MYC (2276S, 1:1000, Cell Signaling Technology), Flag (14793S, 1:1000, Cell Signaling Technology) respectively. For Western blot analysis, except protein examined for Aβ oligomers were resolved on 4−12% Bis‐Tris gels, total cell lysates or concentrated cell supernatant were separated by SDS‐PAGE and blotted on polyvinylidene difluoride membranes (Millipore). The membranes were incubated with specific antibodies to Aβ (15 126, 1:1000, CST), GCA (PA5‐77127, 1:1000, Invitrogen), CCR10 (22071‐1‐AP, 1:1000, Proteintech), Iba‐1 (sc‐32725, 1:500, Santa cruz), APP (60342‐1‐Ig, 1:1000, Proteintech), Presenilin‐1 (16163‐1‐AP, 1:1000, Proteintech), MME (18008‐1‐AP, 1:1000, Proteintech), MMP9 (10375‐2‐AP, 1:1000, Proteintech), CTF (802 803, 1:1000, BioLegend), sAPPα (11 088, 1:1000, IBL), sAPPβ (18 957, 1:1000, IBL), β‐actin (66009‐1‐Ig, 1:5000, Proteintech), and α‐tubulin (11224‐1‐AP, 1:2000, Proteintech), then reprobed with appropriate horseradish peroxidase‐conjugated secondary antibodies. Blots were visualized by enhanced chemiluminescence (ECL Kit; Amersham Biosciences).

### qRT‐PCR Analysis

Briefly, total RNAs were extracted from tissue using AG RNAex Pro Reagent (Accurate Biotechnology (Hunan) Co., Ltd). mRNAs were reverse‐transcribed and then amplified using a real time PCR system (Applied Biosystems). The primer pairs used for qPCR were listed in Table S3 (Supporting Information).

### Cell Migration Assay

Sorted GCA^+^ immune cells and GCA^−^ immune cells were loaded in the upper chamber of a 24‐well transwell plate (8 µm polycarbonate membranes) at 3 × 10^5^ cells in 200 µL DMEM (Gibco) free of FBS. While, different concentration CCL28 protein was added in the bottom chamber including 600 µL DMEM (Gibco) without FBS. After incubation for 24–48 h at 37 °C, cells passing through the filter were fixed and stained using crystal violet for 10 min.

### Membrane and Cytosol Protein Extraction

Cell membrane and cytosol protein extraction was extracted by extraction kit (Beyotime, P0033), according to the manufacturer's instructions. Briefly, collected cells were washed and incubated with reagent A for 15 min. Then, cells were transferred in an ice‐cold Dounce Homogenizer and homogenized on ice for 30–50 times. Cell homogenate was centrifuged at 700 g for 10 min at 4 °C, and collect supernatant and discard the pellet. Supernatant was centrifuged at 14 000 g for 30 min at 4 °C, then collected supernatant was cytosol protein. The pellet was suspended with reagent B, and centrifuged to collect supernatant, which was cell membrane protein. After determination of protein concentration, fluorescent signal of protein was read using a plate reader with an excitation wavelength of 498 nm and emission wavelength of 517 nm.

### Statistical Analyses

The data were expressed as mean ± SEM. The sample size (n) for each statistical analysis was shown in the corresponding figure legends. The Gender data of clinical subjects were analyzed by the Chi‐Square Test. Spearman Correlation Analysis was adopted between the GCA levels and MMSE. The unpaired or paired two‐tailed Student's *t* test was used to compare between two groups. When comparing the difference between multiple groups, one‐way or two‐way ANOVA was applied. All animal experiments were repeated for at least twice. Significance in all Figures is denoted as follows: ^*^ *p* < 0.05, ^**^ *p* < 0.01, ^***^ *p* < 0.001.

## Conflict of Interest

The authors declare no conflict of interest.

## Author Contributions

R.Z. and L.W. contributed equally to this work. H.‐Y.Z. and Y.H. conceived the project and designed the experiments.; R.Z., L.‐W.W., L.‐Y.C, F.X., and P.X. performed the experiments. R.‐Y.Z. and J.W. helped to the data acquisition and analysis. H.‐Y.Z., R.Z. and L.‐W.W. wrote the manuscript. All authors reviewed and revised the manuscript.

## Supporting information

Supporting Information

## Data Availability

The data that support the findings of this study are available from the corresponding author upon reasonable request.
