# Supplementary material for: Bone Marrow‐Derived GCA+ Immune Cells Drive Alzheimer's Disease Progression
Source: Adv Sci (Weinh). 2023 Nov 10;10(36):2303402. doi: 10.1002/advs.202303402 (PMC10754099; doi:10.1002/advs.202303402)
Supplement: Supplementary file 1 — Supporting Information [file ADVS-10-2303402-s001.pdf]

ADVANCED SCIENCE
Open Access

Supporting Information

for *Adv. Sci.*, DOI 10.1002/advs.202303402

Bone Marrow-Derived GCA^+ Immune Cells Drive Alzheimer's Disease Progression

Rui Zhou, Liwen Wang, Linyun Chen, Xu Feng, Ruoyu Zhou, Peng Xiang, Jie Wen, Yan Huang and Haiyan Zhou**

Supplementary Materials for

Bone marrow-derived GCA$^+$ immune cells drive Alzheimer's disease progression

Rui Zhou#, Liwen Wang#, Linyun Chen, Xu Feng, Ruoyu Zhou, Peng Xiang, Jie Wen, Yan Huang *, Haiyan Zhou*

Address all correspondence and request for reprints to:

Prof. Haiyan Zhou,

E-mail: hyzhou02@csu.edu.cn

Prof. Yan Huang,

E-mail: yanhuang1018@csu.edu.cn

The PDF file includes:
Figure S1-S8
Table S1-S3

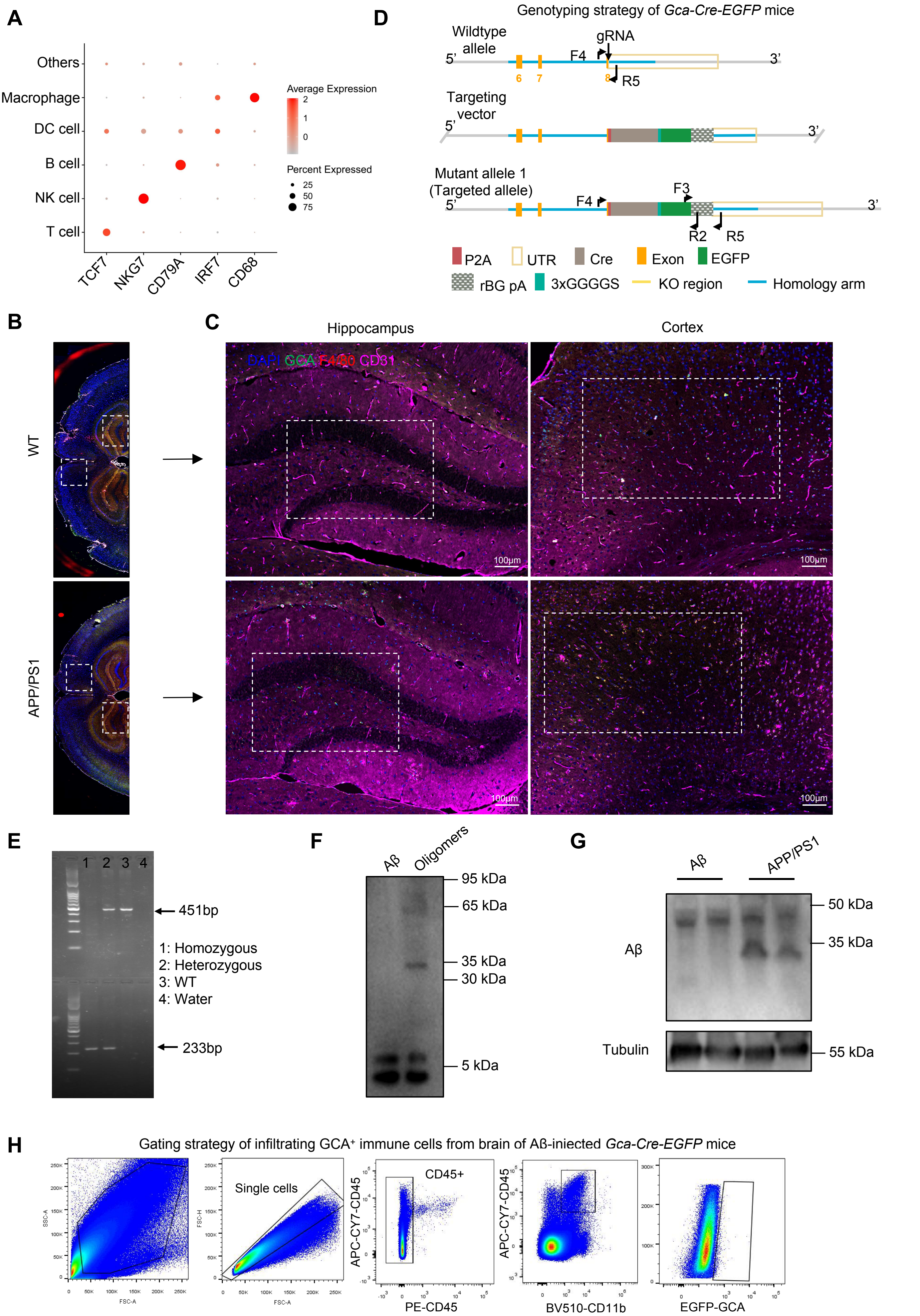

A
Others
Macrophage
DC cell
B cell
NK cell
T cell
TCF7
NKG7
CD79A
IRF7
CD68
Average Expression
2
1
0
Percent Expressed
25
50
75
D
Genotyping strategy of Gca-Cre-EGFP mice
Wildtype allele
gRNA
F4
R5
5'
3'
6
7
8
Targeting vector
Mutant allele 1 (Targeted allele)
F3
R2
P2A
UTR
Cre
Exon
EGFP
rBG pA
3xGGGGS
KO region
Homology arm
B
WT
APP/PS1
C
Hippocampus
Cortex
DAPI GCA F4/80 CD31
100µm
E
1 2 3 4
451bp
1: Homozygous
2: Heterozygous
3: WT
4: Water
233bp
F
Aβ
Oligomers
95 kDa
65 kDa
35 kDa
30 kDa
5 kDa
G
Aβ
APP/PS1
50 kDa
35 kDa
Tubulin
55 kDa
H
Gating strategy of infiltrating GCA⁺ immune cells from brain of Aβ-injected Gca-Cre-EGFP mice
SSC-A
FSC-A
FSC-H
Single cells
CD45+
APC-CY7-CD45
PE-CD45
BV510-CD11b
EGFP-GCA

Figure S1 Generation and validation of *Gca-Cre-EGFP* mice and Aβ oligomers in different AD mouse models.

(A) Dot plots showing the expression levels of specific marker genes for each cell type.

(B) Representative panoramic scan of immunofluorescence staining for GCA (green), F4/80 (red), CD31 (violet), and nuclei (blue). The boxed regions correspond to Figure S1C. (Scale bar = 1000 μm; n = 5-6.)

(C) Representative images of immunofluorescence staining for GCA (green), F4/80 (red), CD31 (violet), and nuclei (blue). The boxed regions correspond to Figure 1D. (Scale bar =100 μm; n=5-6.)

(D) Schematic diagram of breeding strategy for *Gca-Cre-EGFP* mice *in vivo*.

(E) Genotyping result about *Gca-Cre-EGFP* mice.

(F) Representative immunoblots (n = 3 in total) of Aβ oligomer validation in incubation period *in vitro*.

(G) Representative immunoblots (n = 3 in total) of Aβ expression levels in different AD mouse models.

(H) Gating strategy of infiltrating GCA^+ immune cells from brain of Aβ-injected Gca-Cre-EGFP mice.

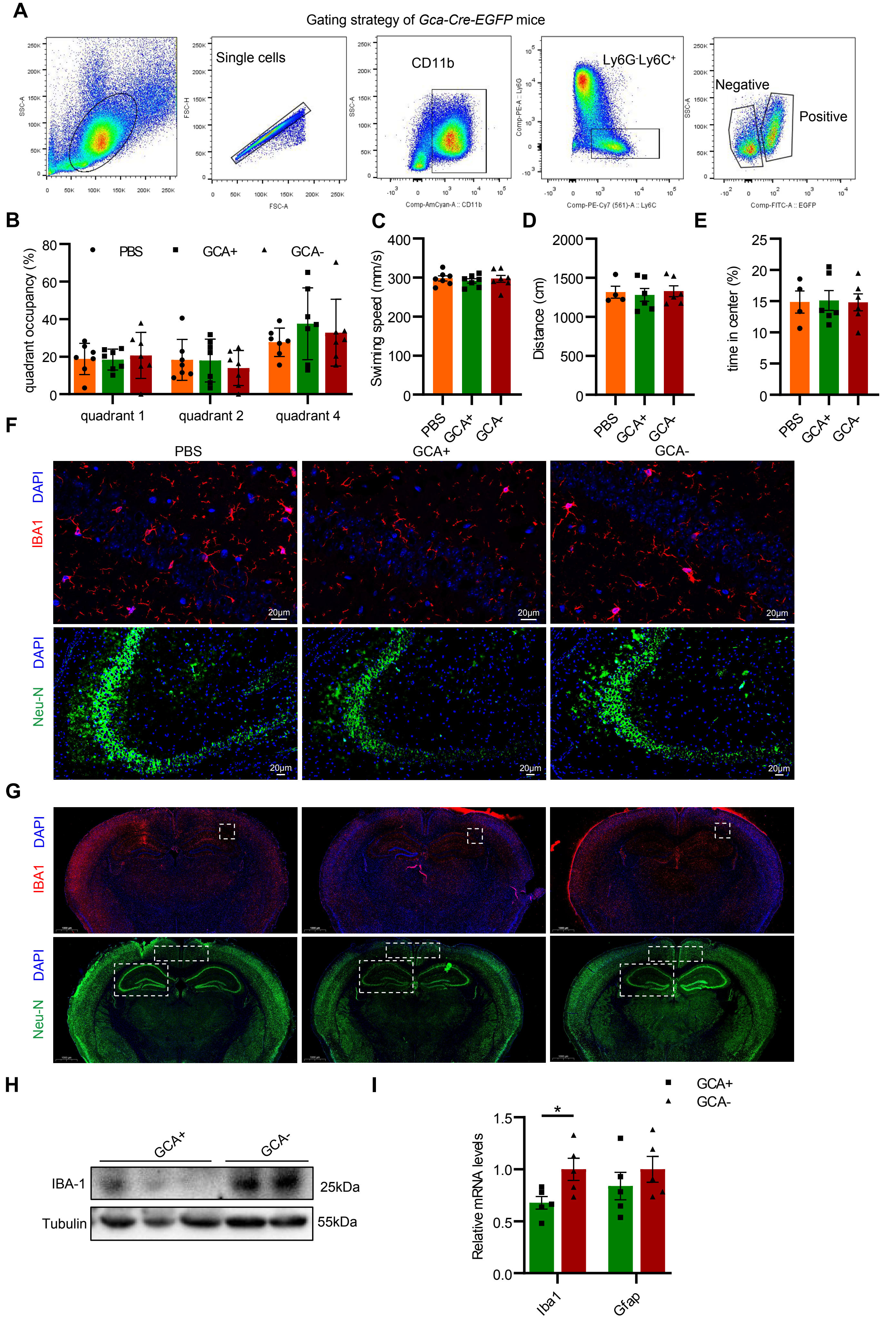
A
Gating strategy of Gca-Cre-EGFP mice
Single cells
CD11b
Ly6G-Ly6C+
Negative
Positive
SSC-A
FSC-H
FSC-A
Comp-AmCyan-A :: CD11b
Comp-PE-A :: Ly6G
Comp-PE-Cy7 (561)-A :: Ly6C
Comp-FITC-A :: EGFP
B
quadrant occupancy (%)
PBS
GCA+
GCA-
quadrant 1
quadrant 2
quadrant 4
C
Swiming speed (mm/s)
D
Distance (cm)
E
time in center (%)
F
PBS
GCA+
GCA-
DAPI
IBA1
Neu-N
20μm
G
H
IBA-1
Tubulin
25kDa
55kDa
I
Relative mRNA levels
Iba1
Gfap
*

Figure S2 Adoptive transfer of bone marrow-derived GCA^+ immune cells suppressed IBA1 expression in AD mouse model.

(A) Gating strategy of sorting GCA^+ immune cells and GCA^- immune cells from *Gca-Cre-EGFP* mice bone marrow cells.

(B) Time spent in other quadrants in GCA^+ immune cells, GCA^- immune cells and PBS control groups (n = 7).

(C) Mean swimming speed of GCA^+ immune cells, GCA^- immune cells and PBS control groups in MWM test (n = 4-6).

(D-E) Total distance traveled (D) and times in center of the open field arena (E) were observed in GCA^+ immune cells, GCA^- immune cells and PBS control groups.

(F) Enlarged picture of Figure 2, I and J (Scale bar = 20 μm)

(G) Representative panoramic scan of immunofluorescence staining for Neu-N (green), IBA1(red), and nuclei (blue). The boxed regions correspond to Figure 2, I ,J and K . (Scale bar = 1000 μm; n = 5.)

(H) Representative immunoblots of IBA1 in GCA^+ immune cells, GCA^- immune cells (n = 2-3).

(I) Relative mRNA levels of *Iba1* and *Gfap* in brains from GCA^+ immune cells, GCA^- immune cells (n = 5).

Data are shown as the mean ± SEM. For panel (B-E): one-way ANOVA. For panel (I):unpaired two-tailed Student's t-test. * $P < 0.05$, ** $P < 0.01$.

A

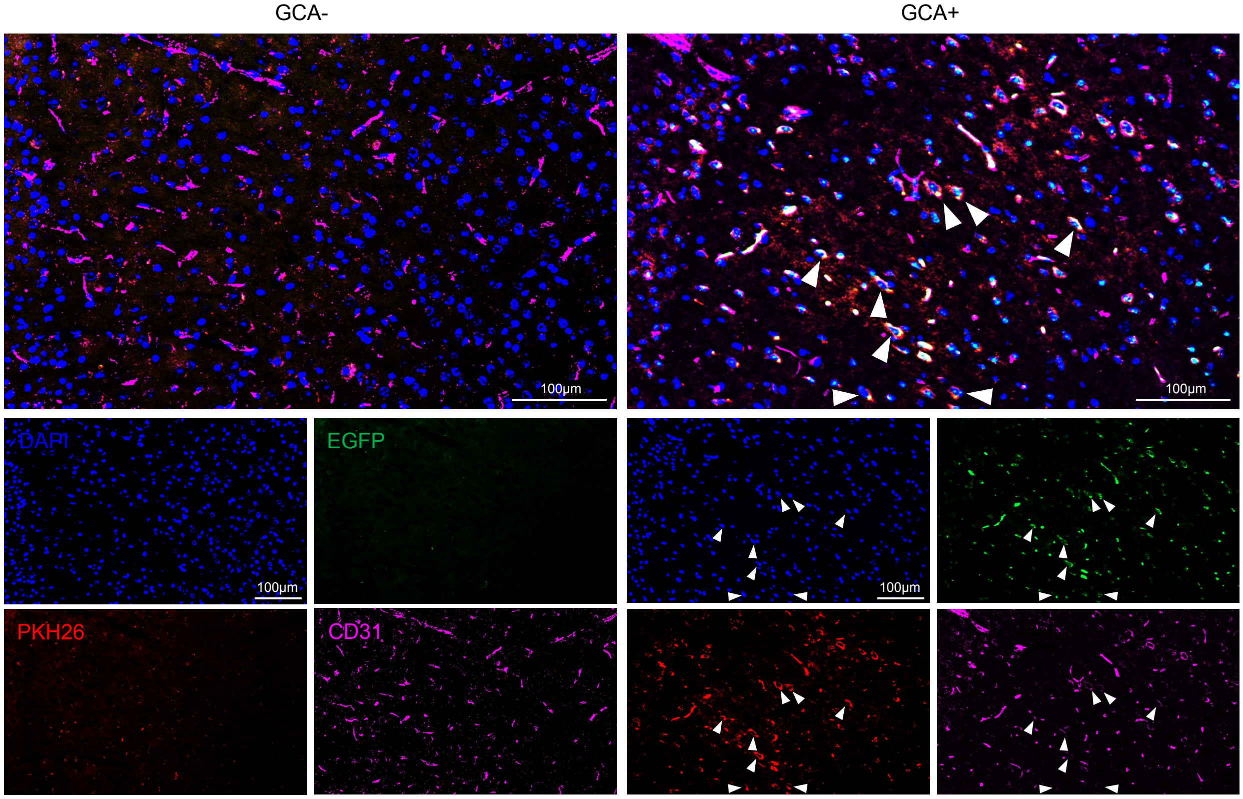

Figure S3 Representative images of transplanted GCA^{+} immune cells infiltrating in cortex.

(A) Representative images of PKH26 (red), EGFP (green), CD31 (violet) and nucleus (blue) staining in cortex from GCA^{+} immune cells, GCA^{-} immune cells groups. White arrows indicate cells within the brain parenchyma. (Scale bar = 100 μm; n = 4).

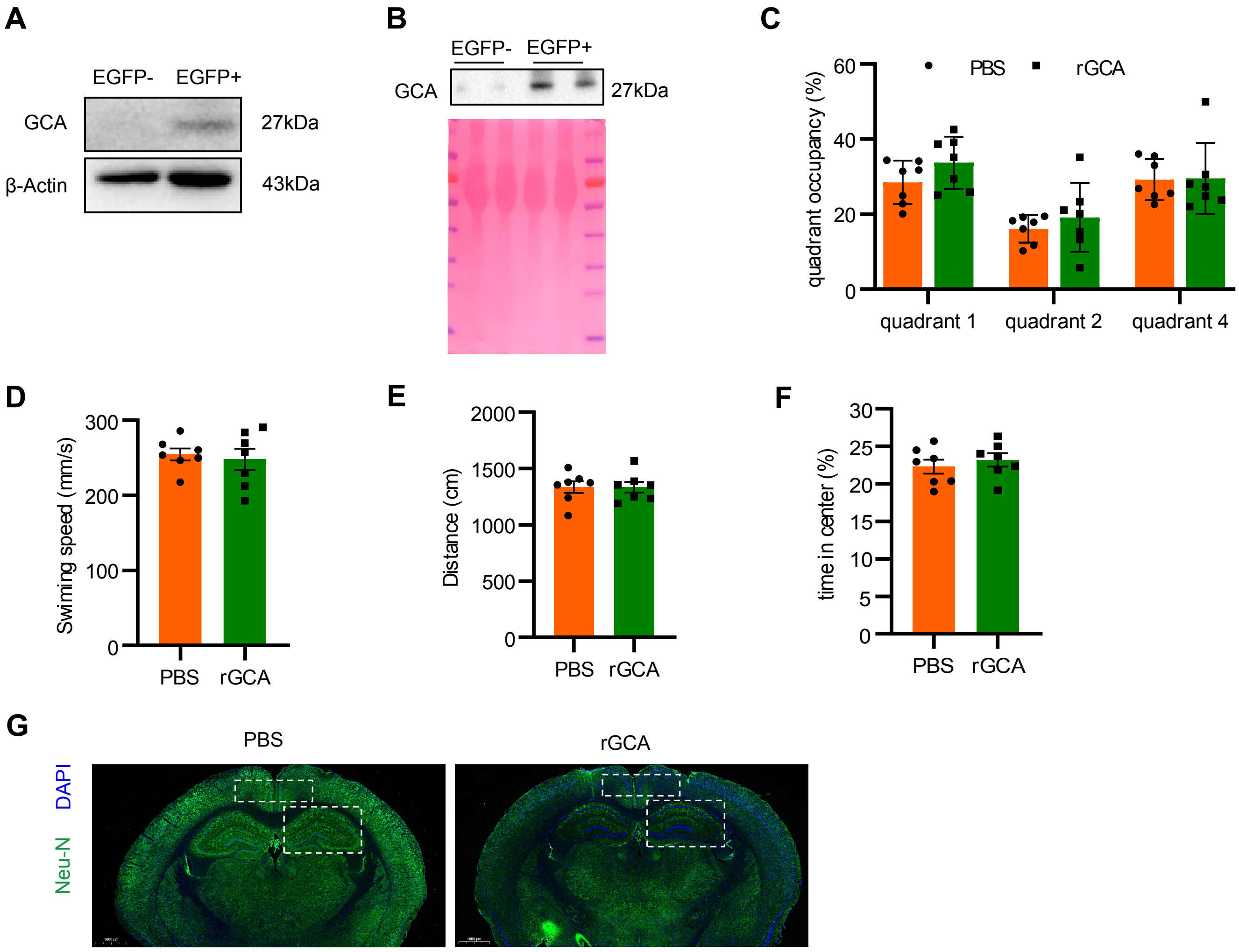

Figure S4 Intracerebral injection of recombinant GCA protein into AD mouse model had no effect on swimming speeds, distance travelled, or time spent in the center.

(A) Representative immunoblots of GCA expression in isolated $EGFP^+$ immune cells and $EGFP^-$ immune cells (n = 3 in total).

(B) Representative immunoblots of GCA expression in the cell culture supernatant of isolated $EGFP^+$ immune cells and $EGFP^-$ immune cells (n = 3 in total).

(C) Time spent in other quadrants in APP/PS1 mice treated with or without rGCA (n = 7).

(D) Mean swimming speed in MWM test in APP/PS1 mice treated with or without rGCA (n = 7).

(E-F) Total distance traveled (E) and time in center of the open field arena (F) were observed in two groups (n = 7).

(G) Representative panoramic scan of immunofluorescence staining for Neu-N (green) and nuclei (blue). The boxed regions correspond to Figure 4L. (Scale bar = 1000 μm; n = 5.)

Data are shown as the mean ± SEM. For panel (B-E): unpaired two-tailed Student's t-test.

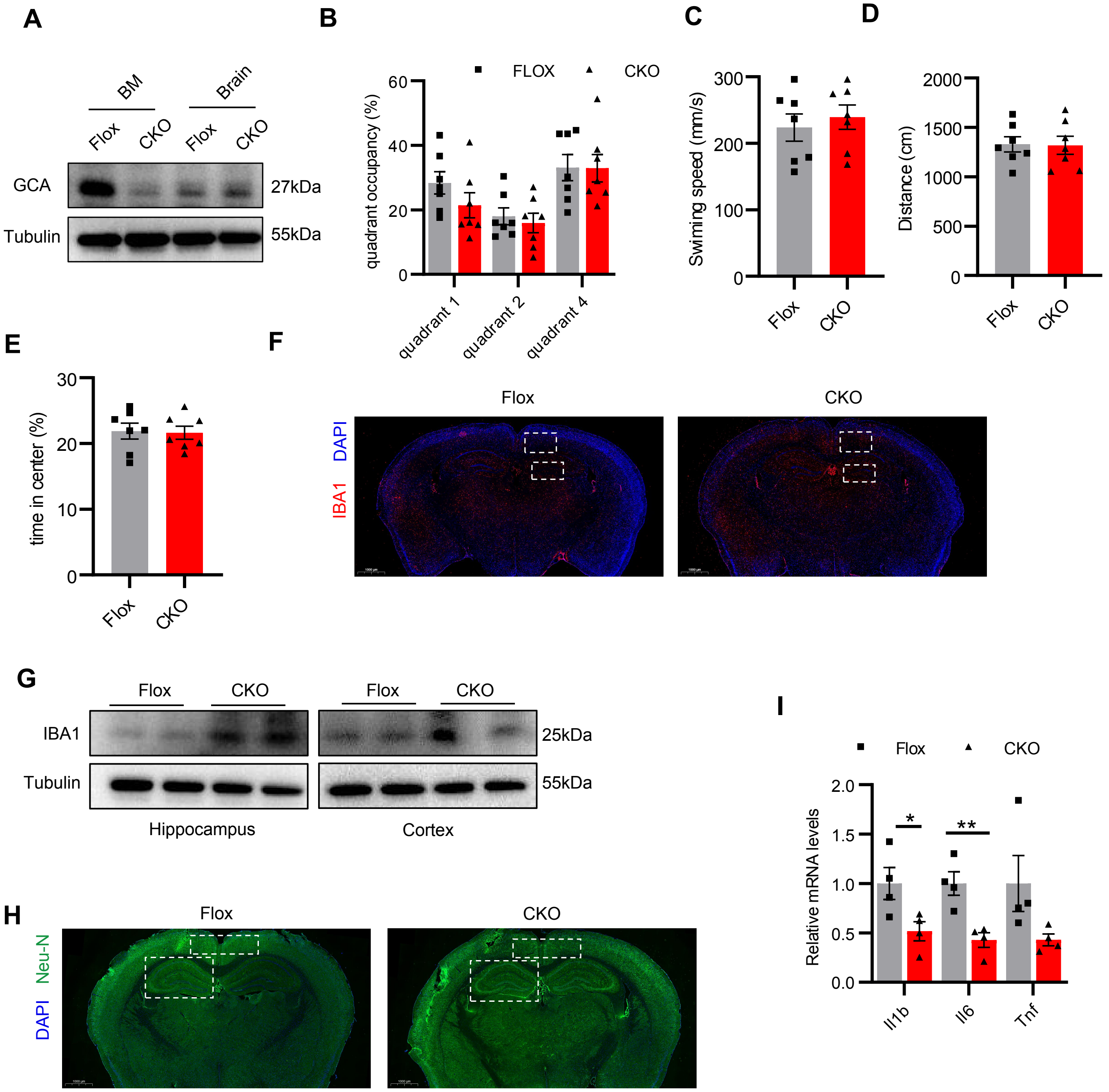
A
BM
Brain
Flox
CKO
Flox
CKO
GCA
27kDa
Tubulin
55kDa
B
FLOX
CKO
quadrant occupancy (%)
60
40
20
0
quadrant 1
quadrant 2
quadrant 4
C
Swiming speed (mm/s)
300
200
100
0
Flox
CKO
D
Distance (cm)
2000
1500
1000
500
0
Flox
CKO
E
time in center (%)
30
20
10
0
Flox
CKO
F
Flox
CKO
DAPI
IBA1
G
Flox
CKO
Flox
CKO
IBA1
25kDa
Tubulin
55kDa
Hippocampus
Cortex
H
Flox
CKO
Neu-N
DAPI
I
Flox
CKO
Relative mRNA levels
2.0
1.5
1.0
0.5
0.0
*
**
Il1b
Il6
Tnf

Figure S5 GCA deficiency in hematopoietic cells enhanced brain IBA1 expression but suppressed neuroinflammation.

(A) Representative immunoblots of GCA in brain and bone marrow from Aβ-injected CKO mice and control mice (n = 3 in total).

(B) Time spent in other quadrants in Aβ-injected CKO mice and control mice (n = 7).

(C) Mean swimming speed in MWM test of Aβ-injected CKO mice and control mice (n = 7).

(D-E) Total distance traveled (D) and time in center of the open field arena (E) were observed in two groups (n = 7).

(F) Representative panoramic scan of immunofluorescence staining for IBA1 (red) and nuclei (blue). The boxed regions correspond to Figure 5, H and I. (Scale bar = 1000 μm; n = 5.)

(G) Representative immunoblots of IBA1 in hippocampus and cortex from Aβ-injected CKO mice and control mice (n = 2).

(H) Representative panoramic scan of immunofluorescence staining for Neu-N (green) and nuclei (blue). The boxed regions correspond to Figure 5, J and K. (Scale bar = 1000 μm; n = 5.)

(I) Relative mRNA level of inflammatory markers in brain from Aβ-injected CKO mice and control mice (n = 4).

Data are shown as the mean ± SEM. For panel (B-E) and (I): unpaired two-tailed Student's t-test. * $P < 0.05$, ** $P < 0.01$.

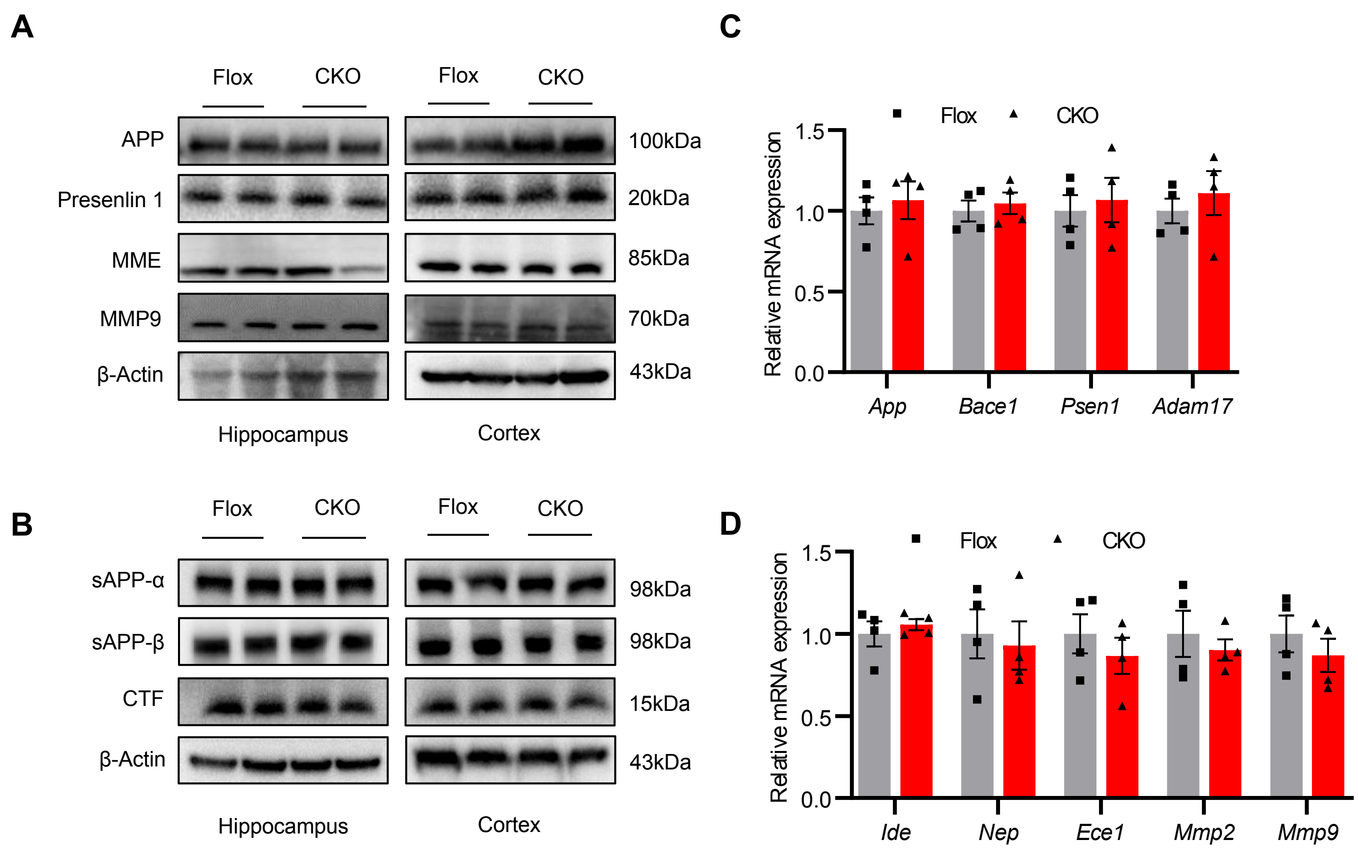

Figure S6 GCA deficiency did not affect APP processing or Aβ degradation.

(A) Representative immunoblots of proteins involving Aβ generation and Aβ degradation in Aβ-injected CKO mice and control mice (n = 5 in total).

(B) Representative immunoblots of proteins involving of APP processing in Aβ-injected CKO mice and control mice (n = 5 in total).

(C)Relative mRNA level of genes about APP processing in Aβ-injected CKO mice and control mice (n = 4).

(D) Relative mRNA level of genes about Aβ degradation in Aβ-injected CKO mice and control mice (n = 4).

Data are shown as the mean ± SEM. For panel (C) and (D): unpaired two-tailed Student’s t-test.

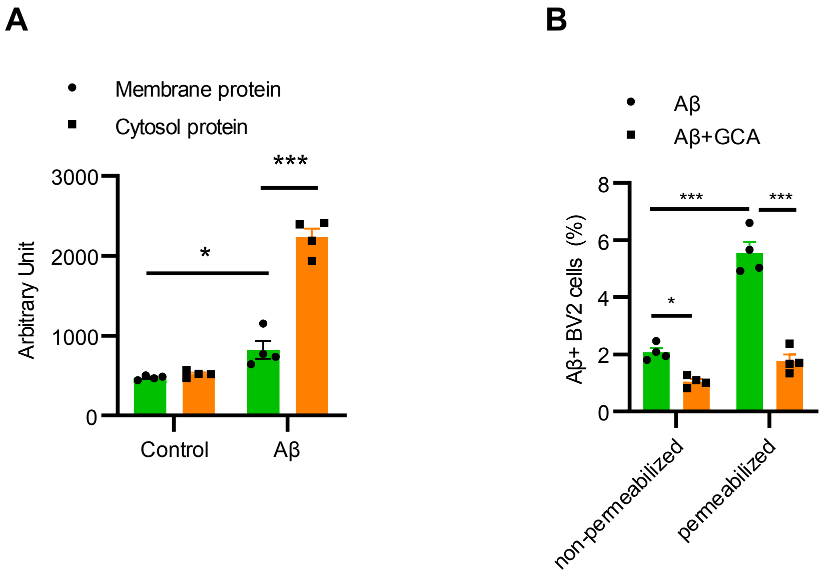

Figure S7 rGCA inhibited Aβ levels both in the cell membrane and cytosol of BV-2 cells.

(A) FITC-Aβ fluorescence intensity of isolated membrane protein and cytosol protein from FITC-Aβ pretreated BV-2 cells (n = 4).

(B) Quantification of the frequencies of BV-2 cells that phagocytose Aβ (n = 4).

Data are shown as the mean ± SEM. Two-way ANOVA with multiple comparison. *, P < 0.05, **, P < 0.01, *** P < 0.001.

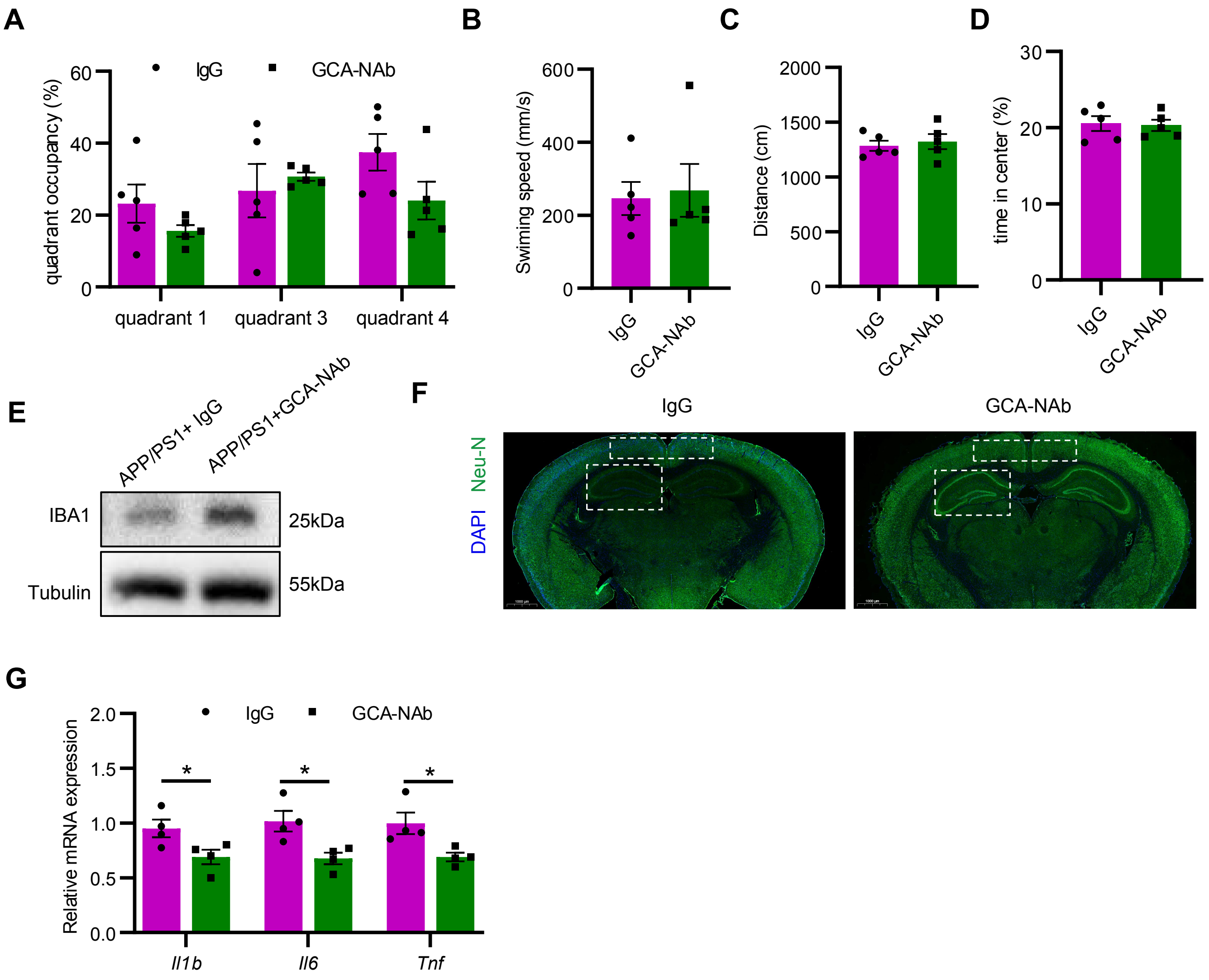

A
quadrant occupancy (%)
IgG
GCA-NAb
quadrant 1
quadrant 3
quadrant 4
B
Swiming speed (mm/s)
C
Distance (cm)
D
time in center (%)
IgG
GCA-NAb
E
APP/PS1+ IgG
APP/PS1+GCA-NAb
IBA1
25kDa
Tubulin
55kDa
F
IgG
GCA-NAb
Neu-N
DAPI
G
Relative mRNA expression
IgG
GCA-NAb
Il1b
Il6
Tnf

Figure S8 GCA neutralizing antibody treatment potentiated microglia IBA1 expression.

(A) Time spent in other quadrants in APP/PS1 mice treated with GCA-NAb or IgG control (n = 5).

(B) Mean swimming speed in MWM test of APP/PS1 mice treated with GCA-NAb or IgG control (n = 5).

(C-D) Total distance traveled (C) and time in center of the open field arena (D) were observed in two groups (n = 5).

(E) Representative immunoblots of IBA1 in APP/PS1 mice treated with GCA-NAb or IgG control (n =3 in total).

(F) Representative panoramic scan of immunofluorescence staining for Neu-N (green) and nuclei (blue). The boxed regions correspond to Figure 7L. (Scale bar = 1000 μm; n = 5.)

(G) Relative mRNA level of inflammatory markers in APP/PS1 mice treated with GCA-NAb or IgG control (n = 4).

Data are shown as the mean ± SEM. For panel (A-D) and (G): unpaired two-tailed Student's t-test.* $P < 0.05$, ** $P < 0.01$.

Table S1. The clinical features of NC and AD subjects.

	NC (n=20)	AD (n=20)	P value
Age (years)	66.20±1.28	65.30±1.71	0.676
Gender			1.000
Male	10 (50%)	10 (50%)	
Female	10 (50%)	10 (50%)	
MMSE	29.20±0.21	13.45±1.00	0.000**
GCA (ng/ml)	5.37±0.09	5.98±0.16	0.002**

Table S2. The clinical features of NC and AD subjects separated by gender.

	Male (n=20)		P value	Female (n=20)		P value
	NC (n=10)	AD (n=10)		NC (n=10)	AD (n=10)	
Age (years)	67.60±1.70	66.10±2.74	0.647	64.80±1.89	64.50±2.17	0.918
MMSE	29.30±0.26	13.80±1.33	0.000**	29.10±0.35	13.10±1.57	0.000**
GCA (ng/ml)	5.40±0.17	6.07±0.24	0.032*	5.35±0.09	5.89±0.21	0.040*

Data were presented as mean ± SEM. Chi-Square test for Gender; unpaired, two-tailed Student's t-test for Age, MMSE, GCA levels. * $p<0.05$ ** $p<0.01$

Table S3 Primer sequences used for real-time PCR

Gene	Primer	Sequence
Mouse *Actb*	Forward primer	GATCATTGCTCCTCCTGAGC
	Reverse primer	ACTCCTGCTTGCTGATCCAC
Mouse *Iba1*	Forward primer	GTCCTTGAAGCGAATGCTGG
	Reverse primer	CATTCTCAAGATGGCAGATC
Mouse *Gfap*	Forward primer	TCCTGGAACAGCAAAACAAG
	Reverse primer	CAGCCTCAGGTTGGTTTCAT
Mouse *Ccl28*	Forward primer	TCATTGCCAGACTCAGTGGG
	Reverse primer	GCCATGGGAAGTATGGCTTCT
Mouse *Il1b*	Forward primer	GCCACCTTTTGACAGTGATGAG
	Reverse primer	AAGGTCCACGGGAAAGACAC
Mouse *Il6*	Forward primer	CAATGGCAATTCTGATTGTATG
	Reverse primer	AGGACTCTGGCTTTGTCTTTC
Mouse *Tnf*	Forward primer	CCCTCACACTCAGATCATCTTCT
	Reverse primer	GCTACGACGTGGGCTACAG
Mouse *App*	Forward primer	AGAGGTCTACCCTGAACTGC
	Reverse primer	AGAGGTCTACCCTGAACTGC
Mouse *Bace1*	Forward primer	AGAGGTCTACCCTGAACTGC
	Reverse primer	AGGATGGTGATGCGGAAG
Mouse *Psen1*	Forward primer	TGAGGAGGAAGACGAAGA
	Reverse primer	CACGGCGACATTGTAGGA
Mouse *Adam17*	Forward primer	TGTGAGCGGTGACCACGAGAAT
	Reverse primer	TTCATCCACCCTGGAGTTGCCA
Mouse *Ide*	Forward primer	AATCCGGCCATCCAGAGAATA
	Reverse primer	GGGTCTGACAGTGAACCTATGT
Mouse *Nep*	Forward primer	CTCTCTGTGCTTGTCTTGCTC
	Reverse primer	GACGTTGCGTTTCAACCAGC
Mouse *Ece1*	Forward primer	CAGGTGGTCACAGCTCACTAC
	Reverse primer	GGTATCCAGTCAGGACCTTTTCA
Mouse *Mmp2*	Forward primer	CAAGTTCCCCGGCGATGTC
	Reverse primer	TTCTGGTCAAGGTCACCTGTC
Mouse *Mmp9*	Forward primer	GCAGAGGCATACTTGTACC
	Reverse primer	TGATGTTATGATGGTCCCACTTG
